# The Dietary Inflammatory Index and Incident Risk of Type 2 Diabetes Mellitus: Interactions with Obesity and Dyslipidemia in a Prospective Cohort Study

**DOI:** 10.3390/nu18050738

**Published:** 2026-02-25

**Authors:** Jinliang Liang, Xueru Fu, Yuying Wu, Taifeng Chen, Yaqin Su, Li Yang, Minqi Gu, Liuding Wen, Yang Zhao, Kexin Li, Yihao Shu, Kaixin Chen, Jinyuan Pang, Dongsheng Hu, Ming Zhang

**Affiliations:** 1 Department of Biostatistics and Epidemiology, School of Public Health, Shenzhen University Medical School, Shenzhen 518060, China; 2 Department of Epidemiology and Biostatistics, College of Public Health, Zhengzhou University, Zhengzhou 450001, China

**Keywords:** dietary inflammatory index, obesity, dyslipidemia, interaction, T2DM, cohort study

## Abstract

Objectives: To explore the association between the dietary inflammatory index (DII) and type 2 diabetes mellitus (T2DM) risk and to evaluate potential interactions of obesity and dyslipidemia in the context of this association. Methods: This cohort study included 8055 adults. Dietary data from food frequency questionnaires were used to calculate DII, reflecting dietary inflammatory potential. T2DM was defined as fasting plasma glucose ≥7.0 mmol/L, HbA1c ≥6.5%, a documented T2DM history, or glucose-lowering therapy. Multivariate Cox regression models assessed the DII-T2DM association, with multiplicative interaction analysis via product terms and additive interactions evaluated using relative excess risk due to interaction (RERI) and attributable proportion due to interaction (AP). Results: After a median 5.01-year follow-up, 1034 incident T2DM cases had occurred. The highest versus lowest DII quartile showed an unadjusted HR of 1.20 (95% CI: 1.01–1.42), which attenuated after adjusting for demographic and clinical confounders. In women, the highest DII quartile had a significantly adjusted HR of 1.36 (1.03–1.81), with a 16% increased risk per 1-SD DII increase (adjusted HR:1.16, 95% CI:1.04–1.29); no association was observed in men. Positive multiplicative and additive interactions emerged in total participants between high DII and central obesity (measured by waist circumference/waist-to-hip ratio), accounting for 22% and 31% of excess T2DM risk, respectively. No interactions were found with dyslipidemia and other obesity metrics (BMI, waist-to-height ratio). Conclusions: This study suggests that a highly pro-inflammatory diet may be associated with an increased incident risk of T2DM among women. Waist circumference and waist-to-hip ratio and a high DII are found to act synergistically in elevating T2DM risk.

## 1. Introduction

The latest data show that the global incidence of diabetes in 2024 was 589 million, with 98% of cases being type 2 diabetes mellitus (T2DM). Projections suggest that this global prevalence will surge to 853 million by 2050 [1]. China bears the highest diabetes burden, witnessing a 56% rise in cases (90 to 141 million) over the past decade, reaching 148 million cases in 2024, and expected to reach 168 million by 2050 [1]. As highlighted in the Diabetes Atlas (11th Edition, 2025), this escalating epidemic underscores an urgent need for targeted prevention strategies through risk factor identification.

Chronic low-grade inflammation serves as a key mechanistic driver in diabetes development, intimately connected with insulin resistance [2] and β-cell dysfunction [3]. Studies have shown that diet can influence diabetes development through modulation of inflammatory responses [4,5]. The dietary inflammatory index (DII), developed by Shivappa and colleagues [6], quantifies the inflammatory potential of habitual diets based on associations between nutrients and inflammatory markers; it has been validated across populations [7,8]. Previous studies using the DII suggest pro-inflammatory diets may increase T2DM risk [9,10]. The results reported in the pertinent literature remain inconsistent, however, with other studies reporting no association between higher DII and T2DM risk [11]. Moreover, accumulating evidence suggests sex-specific associations between dietary inflammatory potential and T2DM risk. Several biological pathways may explain these differences. First, sex steroid hormones, particularly estrogen, exert anti-inflammatory effects and may help protect against insulin resistance [12,13]. The decline in estrogen during menopause is linked to increased systemic inflammation and metabolic dysfunction [14], potentially heightening women’s susceptibility to pro-inflammatory diets. Second, sex differences in body composition and fat distribution may modulate the relationship between dietary inflammation and diabetes risk [15]. Third, several epidemiological studies have reported stronger DII–diabetes associations in women than in men [16,17,18], though the mechanisms remain unclear. Additionally, sex differences in gut microbiome composition and immune regulation may differentially mediate the diet–inflammation–diabetes pathway [19]. These observations support the need for sex-stratified analyses when examining DII-T2DM associations. The generalizability across diverse populations remains questionable, however. To our knowledge, existing research has predominantly focused on Western populations [11], while evidence from Chinese populations, especially those in low-income rural areas with monotonous diets, is scarce. Further, most studies are cross-sectional [20], lacking prospective evidence to establish causality. Prospective studies in diverse populations with sex-stratified analyses are therefore needed to clarify the causal role of dietary inflammation in T2DM etiology.

Obesity, a major T2DM risk factor, also drives chronic inflammation [21]. The E3N cohort study found a multiplicative interaction between BMI and DII in relation to T2DM risk, suggesting that adiposity may modify the effect of dietary inflammation on T2DM [17]; however, other prospective cohort studies found no significant interaction between DII and obesity measures [22,23], including no interaction between E-DII and obesity in the Women’s Health Initiative (*p* = 0.73) [22]. These inconsistent findings highlight the need for further investigation using comprehensive obesity metrics and both multiplicative and additive interaction models. Additionally, dyslipidemia shares inflammatory pathways with dietary patterns [24] and may modulate associations between DII and metabolic outcomes, as observed for cardiovascular disease [25]. Whether dyslipidemia influences the DII-T2DM association remains unknown and warrants investigation. Further, evidence regarding sex-specific potential effect modification remains limited, particularly in populations outside Western contexts [20].

This study, therefore, aims to investigate the association between the DII and the risk of incident T2DM, concurrently analyze potential sex-specific differences in this association, and explore the interactions of diverse obesity metrics and dyslipidemia subtypes with DII in relation to T2DM risk, using data from the Rural Chinese Cohort Study (RCCS). These analyses seek to elucidate the complex association of dietary inflammation, obesity, and dyslipidemia in T2DM pathogenesis, with the aim of providing scientific evidence for precision-targeted T2DM prevention and control strategies.

## 2. Methods

### 2.1. Study Design and Participants

The RCCS is a large-scale ongoing prospective cohort study. A total of 20,194 adults from rural Henan Province in China were enrolled through cluster random sampling during baseline surveys conducted in 2007–2008. The first follow-up round was completed in 2013–2014 (response rate: 85.50%, *n* = 17,265), with the second round being conducted in 2018–2020 (response rate: 92.86%, *n* = 18,752). Detailed methodologies for the cohort establishment and data collection have been described previously [26].

This study utilized follow-up data collected during the 2013–2014 and 2018–2020 survey periods. Follow-up person-years were calculated from the first RCCS follow-up to the second, or to T2DM diagnosis or death. Of the 17,265 participants who completed the 2013–2014 follow-up, 1110 were identified as deceased at the time of contact, leaving 16,155 participants with active follow-up data available for this analysis. The following participants were further excluded: (1) participants diagnosed with diabetes at the 2013–2014 follow-up (*n* = 2167); (2) participants with malignant tumors, renal failure, or end-stage renal disease at the 2013–2014 follow-up (*n* = 68); (3) participants without dietary data in the 2013–2014 follow-up (*n* = 3759); and (4) participants with unknown T2DM status at the 2018–2020 follow-up (*n* = 2106). Ultimately, data from 8055 eligible participants were incorporated into this analysis. Figure S1 is a comprehensive flowchart of the sample-choosing procedure. Every participant provided written informed consent, while Shenzhen University’s Medical Ethics Committee accepted the study’s procedure.

### 2.2. Dietary Assessment and Calculation of DII

Dietary assessment was undertaken using a validated food frequency questionnaire (FFQ) capturing 12-month retrospective consumption patterns. Intake frequencies were categorized into quintile intervals (daily, weekly, monthly, annual, never). Daily nutrient intake was derived through the integration of participant-reported food consumption frequencies and portion sizes, utilizing the 6th Edition of the Standardized Chinese Food Composition Tables [27].

The DII was developed via standardized associations between 45 food parameters (comprising three categories: nutrients, bioactive compounds, and food items) and six key inflammatory biomarkers, interleukin (IL)-1β, tumor necrosis factor (TNF)-α, IL-4, IL-6, IL-10, and C-reactive protein (CRP), generating a composite score to assess the diet’s overall anti-inflammatory or pro-inflammatory potential. The detailed development, calculation method, and validation of the DII are described in previously published references [6,28]. In our study, Individual DII scores were calculated using 24 out of 45 available food parameters. More negative DII scores indicate better anti-inflammatory effects of the diet, while more positive DII scores indicate stronger pro-inflammatory effects. The detailed calculation steps of DII in this study are shown in Table S1.

### 2.3. Covariate Assessment

Trained researchers used standardized questionnaires to collect socio-demographic data (age, gender, educational level, marital status, and average monthly income), lifestyle/behavioral factors (smoking status, drinking status, physical activity, and sleep duration), reproductive information (menopausal status and pregnancies for female participants), medical histories, and family disease histories. Educational level was categorized as high school and above or below high school level; marital status as married/cohabiting or other; and average monthly income as <1000 CNY, 1001–3000 CNY, or ≥3001 CNY. Smoking status was classified as never smoked, current smoking, or quit smoking, with current smoking defined as currently smoking and/or having smoked at least 100 cigarettes in a lifetime [29]. Alcohol drinking was classified as yes (≥12 drinks in the past year) or no [29]. Sleep duration was categorized as short (<7 h), normal (7–8 h), or long (≥8 h) [30]. For female participants, menopausal status was classified as premenopausal or postmenopausal, and number of pregnancies was recorded. Physical activity was classified as ideal (≥150 min of moderate or ≥75 min of vigorous activity per week) or non-ideal based on the International Physical Activity Questionnaire [31] measured in metabolic equivalents [32,33]. Diabetes family history was defined as one or more first-degree relatives having diabetes.

Anthropometric measurements (weight: to 0.5 kg precision; height, waist/hip circumference: to 0.1 cm precision) were obtained following standardized protocols. Body mass index (BMI) was calculated as weight (kg)/height (m)^2^. Waist-to-hip ratio (WHR) was calculated as waist circumference (WC) (cm)/hip circumference (cm), and waist-to-height ratio (WHtR) as WC (cm)/height (cm). Blood pressure was measured three times at 30-s intervals using an electronic sphygmomanometer. Hypertension definition [34] was systolic/diastolic blood pressure ≥140/90 mmHg or the administration of antihypertensive medicines. Fasting blood samples (≥8 h) were analyzed for fasting plasma glucose (FPG), total cholesterol (TC), triglycerides (TG), and high-density lipoprotein cholesterol (HDL-C) by an automated biochemical analyzer, with low-density lipoprotein cholesterol (LDL-C) calculated via the Friedewald formula [35]. 

### 2.4. Assessment of Obesity and Dyslipidemia

Obesity was defined by the following criteria: (1) BMI ≥ 28 kg/m^2^ [36]; (2) WC ≥ 90 cm for men and ≥85 cm for women [36]; (3) WHR ≥ 0.90 for men and ≥0.85 for women [37]; or (4) WHtR ≥ 0.5 [38]. According to the “Chinese Guidelines for the Prevention and Treatment of Dyslipidemia in Adults” [39], hypercholesterolemia (high TC) was defined as TC ≥ 6.22 mmol/L, hypertriglyceridemia (high TG) as TG ≥ 2.26 mmol/L, low HDL-C as HDL-C < 1.04 mmol/L, and high LDL-C as LDL-C ≥ 4.14 mmol/L. Dyslipidemia was defined as TC ≥ 6.22 mmol/L and/or TG ≥ 2.26 mmol/L and/or HDL-C < 1.04 mmol/L and/or LDL-C ≥ 4.14 mmol/L.

### 2.5. Diagnostic Criteria for T2DM

The outcome event of this study is T2DM. The following criteria define T2DM [40]: self-reported history of T2DM, glucose-lowering therapy, FPG ≥ 7.0 mmol/L, or HbA1c ≥ 6.5%, excluding type 1 diabetes mellitus, gestational diabetes mellitus, and other special types of diabetes mellitus.

### 2.6. Statistical Analysis

In our analysis, normality tests revealed skewed distributions in continuous variables, so they were expressed as median (interquartile range, IQR) and analyzed with Mann–Whitney U/Kruskal–Wallis H tests; categorical variables were summarized as frequency (percentage) and assessed by chi-square tests.

Based on DII quartiles, the study subjects were split into four groups (Q1–Q4). Cox regression models assessed hazard ratio (HR) and 95% CI for T2DM risk between DII categories (with the lowest quartile as reference) and for each 1-standard deviation (SD) increase in DII. To better identify gender differences, separate analyses were conducted for all participants, male and female. Potential confounders were selected based on previous studies, and three models were constructed: Model 1 was unadjusted; Model 2 was adjusted for age, gender, educational level, marital status, and average monthly income; and Model 3 included adjustments for the variables in Model 2, as well as smoking status, drinking status, PA, sleep duration, BMI status, family history of diabetes, energy intake, hypertension, dyslipidemia and fasting plasma glucose. Variance inflation factor (VIF) analysis confirmed no substantial multicollinearity (all VIF < 5) among the covariates included in our models (Table S2). Subgroup analyses, stratified by age, alcohol drinking, smoking status, ideal PA, hypertension and family history of diabetes, were conducted to examine the heterogeneity across different populations, then the interaction between these stratification variables and DII was assessed by including their product terms in the Cox models. Dose–response associations were modeled using restricted cubic splines at the 10th, 50th, and 90th DII percentiles.

In addition, to assess the potential effect modification of obesity and dyslipidemia on the DII-T2DM association, we dichotomized DII into high-inflammatory (Q4) and low-inflammatory (Q1–Q3) diet groups. This categorization was based on the recommended methodology for calculating additive interaction measures in proportional hazards models [41,42,43], consistency with previous DII-related interaction studies [44], and the study participants’ generally elevated dietary inflammatory levels (median DII = 2.58), where Q4 represents individuals with the most extreme pro-inflammatory dietary patterns. The joint association of high DII levels, different obesity criteria, and types of dyslipidemia on T2DM risk was assessed using a fully adjusted model, with low-DII individuals with normal lipid levels and no obesity as the reference group. Subsequently, we assessed the effect modifications on multiplicative and additive scales using the fully adjusted Cox models. The HR with its 95% CI for the interaction term was the measure of interaction on the multiplicative scale, obtained by dividing the observed joint effect by the product of the independent (isolated) effects. When HR > 1 and 95% CI excluded 1, it was regarded as a positive multiplicative interaction. Otherwise, it was negative [45]. The relative excess risk due to interaction (RERI) and the attributable proportion due to interaction (AP) and their corresponding 95% CIs were computed to examine the additive interaction. Additive interaction was indicated when 95% CI of RERI and AP did not include 0. A positive interaction existed if RERI > 0 and AP > 0; it was the opposite for a negative interaction [42,46]. Additionally, to explore potential gender-specific effect modifications, all multiplicative and additive interaction analyses were repeated separately for male and female participants, using the same fully adjusted Cox models and reference groups as the overall population analysis.

To assess the robustness of the DII-T2DM risk association, we performed four sensitivity analyses. First, to rule out potential reverse causation, we excluded participants diagnosed with T2DM within the first two years of follow-up. Second, to account for missing covariate data, we implemented multiple imputation via chained equations (MICE) for all incomplete covariates. Third, to examine whether the observed associations were sensitive to the choice of adiposity measure, we conducted additional analyses in which BMI in Model 3 was substituted with alternative central obesity metrics (WHtR, WHR, or WC). Additionally, given that menopausal status and pregnancies may affect systemic inflammation and insulin resistance in women, we performed a female-specific sensitivity analysis by additionally adjusting for these factors in Model 3 to verify the robustness of the DII-T2DM association in women.

Statistical analyses were conducted using either SAS 9.4 (SAS Institute Inc., Cary, NC, USA) or R 4.4.2 (R Foundation for Statistical Computing, Vienna, Austria). A two-tailed *p* < 0.05 was considered statistically significant.

## 3. Results

### 3.1. Baseline Characteristics of Study Participants

This study enrolled 8055 participants with an average age of 56 (IQR: 47–, 65), including 3241 males (40.24%) and 4814 females (59.76%). Over a median follow-up of 5.01 years, 1034 incident cases of T2DM were identified. The DII scores of all participants ranged from −3.86 to 4.33. Table 1 shows the baseline characteristics of study participants stratified by T2DM status. Compared with non-T2DM individuals, T2DM patients were older, had lower education levels and physical activity, higher adiposity metrics (BMI, WC, WHtR, WHR), elevated systolic/diastolic pressure, and adverse lipid profiles (increased TC, TG, LDL-C; decreased HDL-C) (all *p* < 0.05). Baseline characteristics stratified by DII quartiles and gender are presented in Tables S3 and S4, respectively. A comparative analysis of food parameter intakes contributing to DII scores showed no significant differences for most nutrients (all *p* > 0.05); however, T2DM patients had lower intakes of anti-inflammatory nutrients (PUFA, isoflavones, anthocyanins) and higher carbohydrate intakes (all *p* < 0.05) (Table S5). Corresponding food parameter-specific DII scores grouped by overall DII quartiles are presented in Table S6.

### 3.2. Association of DII with the Risk of T2DM

To evaluate the association between DII and the risk of T2DM, Cox proportional hazards regression analyses were performed (Table 2). Among the total participants, the unadjusted model showed elevated T2DM risk in the highest DII quartile (Q4) relative to the lowest (Q1) (HR = 1.20, 95% CI:1.01–1.42; *p* = 0.038), and with each 1-SD increase in DII (HR = 1.07, 95% CI:1.01–1.13; *p* = 0.030). Both associations were attenuated to non-significance after full adjustment (Model 3).

**Figure 1:**
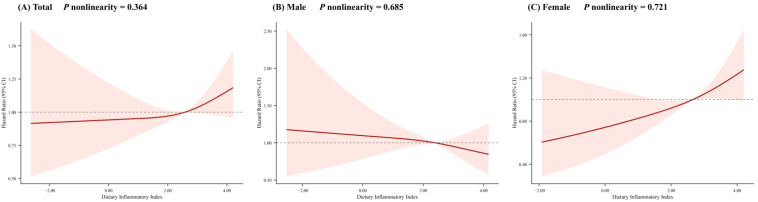
Dose–response association of DII with the risk of T2DM. (**A**) in total participants; (**B**) in male participants; (**C**) in female participants. Adjusted for age, gender, educational level, marital status, average monthly income, smoking status, drinking status, physical activity, sleep duration, BMI status, family history of diabetes, energy intake, hypertension, dyslipidemia, and fasting plasma glucose.

Gender stratification revealed divergent effects (Table 2). No significant associations were observed in males across all models. Conversely, among females, participants in Q4 had an elevated risk of T2DM compared to Q1 in Model 1 (HR = 1.48, 95% CI:1.17–1.85, *p* = 0.001). As a continuous variable, each 1-SD increase in DII was associated with a 17% higher risk in Model 1 (HR = 1.17, 95% CI:1.08–1.27, *p* < 0.001). This association remained significant after full adjustment (Model 3), with HR for Q4 (1.36, 95% CI:1.03–1.81, *p* = 0.031) and each 1-SD DII (1.16, 95% CI:1.04–1.29, *p* = 0.008). A linear association between DII and T2DM risk was observed (*P* nonlinearity > 0.05) (Figure 1). These results indicate that the association between DII and T2DM risk was stronger in women, highlighting significant gender differences (*P*_interaction-by-gender_ = 0.025).

### 3.3. Subgroup Analysis for DII and the Risk of T2DM

Subgroup analyses were conducted to examine the DII-T2DM association across various socio-demographic characteristics and lifestyle factors (Table S11). No statistically significant interaction was found for any covariates (all *p* for interaction > 0.05), including age, alcohol drinking, smoking status, ideal physical activity, hypertension, or family history of diabetes. When DII was analyzed as a continuous variable, a modest increase in T2DM risk per 1-SD increase was observed among non-drinkers (HR = 1.10, 95% CI:1.01–1.20, *p* = 0.034) and never smokers (HR = 1.11, 95% CI:1.00–1.23, *p* = 0.045). No such association was seen in their counterpart subgroups (such as drinkers or current smokers), and no categorical (quartile) comparisons reached significance. In summary, the association observed in the fully adjusted model for the overall population was generally consistent across subgroups.

### 3.4. Interactions and Joint Associations of Obesity and Dyslipidemia with DII on T2DM Incident Risk

For total participants, Table 3 shows the interaction and joint associations of obesity metrics on the association between binary DII groups and risk of T2DM. Joint-association analyses show that among individuals classified as obese by WHR criteria, those with a high-inflammatory diet (high DII levels) had a 1.50-fold increased T2DM risk (95% CI: 1.19–1.90, *p* = 0.001) compared with non-obese individuals with a low-inflammatory diet. Significant positive multiplicative interaction (HR for multiplicative:1.62, 95% CI: 1.08–2.44, *p* = 0.020) and additive interaction (RERI = 0.47, 95% CI: 0.07–0.80) were observed between high DII and WHR-defined obesity, with the interaction accounting for 31% of T2DM risk (AP = 0.31, 95% CI: 0.05–0.52). Similarly, individuals defined as obese by WC criteria and with high DII had a 1.84-fold increased risk of T2DM (95% CI: 1.45–2.33, *p* < 0.001) compared with non-obese individuals with low DII scores. Positive multiplicative interaction (HR for multiplicative:1.39, 95% CI: 1.02–1.90, *p* = 0.038) and additive interaction (RERI = 0.41, 95% CI: 0.02–0.81) were observed, with the interaction between high DII and WC-defined obesity contributing 22% to the risk of T2DM (AP = 0.22, 95% CI: 0.00–0.39). Obese individuals by WHtR criteria with high DII had a 1.46-fold increased T2DM risk (95% CI: 1.13–1.90), but no significant interactions were observed for effect modification on multiplicative or additive scales. No significant joint association or multiplicative/additive interactions were observed between high DII and BMI-defined obesity. In addition, we failed to observe significant joint associations or multiplicative/additive interactions between high DII and any type of dyslipidemia (Table 4).

To further explore sex-specific potential effect modifications, we conducted sex-stratified analyses. Among males (Table S7), significant multiplicative interactions were observed between high DII and obesity defined by BMI (HR for multiplicative: 2.12, 95% CI: 1.13–3.98, *p* = 0.019), WHR (HR for multiplicative: 2.14, 95% CI: 1.09–4.18, *p* = 0.027), and WC (HR for multiplicative: 2.41, 95% CI: 1.34–4.34, *p* = 0.003). A significant additive interaction was found for WC-defined obesity (RERI = 0.83, 95% CI: 0.11–1.76; AP = 0.41, 95% CI: 0.02–0.62). In terms of joint association, males with WC-defined obesity and high DII had a significantly higher T2DM risk (HR = 2.03, 95% CI: 1.33–3.09, *p* = 0.001) compared to the reference group (normal WC and low DII). Among females (Table S9), the pattern differed. No significant multiplicative or additive interactions were found between high DII and any obesity metrics; however, significant joint associations were observed: females with WHR-defined obesity and high DII had an increased T2DM risk (HR = 1.43, 95% CI: 1.03–1.98, *p* = 0.033), and those with WC-defined obesity and high DII also had an elevated risk (HR = 1.80, 95% CI: 1.33–2.43, *p* < 0.001), both compared to their respective non-obese, low-DII reference groups. Regarding dyslipidemia, no significant joint associations or interactions between high DII and any of the examined types (high TC, high TG, low HDL-C, or high LDL-C) were observed in either males or females (Tables S8 and S10).

### 3.5. Sensitivity Analysis

To assess the robustness of our findings, we performed four sensitivity analyses. Excluding individuals who developed T2DM within the first two years of follow-up did not alter the observed associations (Table S12). Similarly, after multiple imputation of missing covariates, the association between DII and T2DM risk remained consistent with the primary results (Table S13). Substituting BMI with alternative adiposity metrics (WHtR, WHR, or WC) in Model 3 also yielded largely consistent results (Table S14). Moreover, among female participants with further adjustment for menopausal status and pregnancies, the DII-T2DM association remained robust (Table S15). All four sensitivity analyses confirmed the sex-specific pattern, with significant positive associations between higher DII scores and T2DM risk in women, but not in men.

## 4. Discussion

This study, based on data from a prospective cohort among the rural population of China, investigated the association between DII and T2DM risk and further evaluated the interaction of DII with different obesity metrics and dyslipidemia types. Our results indicate a gender difference in the association between DII and T2DM, with a positive association observed in women, whereas no association emerged in men. Additionally, we found positive multiplicative and additive interactions between DII and specific central obesity metrics (WC and WHR) in total participants, but not with dyslipidemia or other obesity metrics (BMI and WHtR).

The modified DII score formulated by Shivappa and colleagues [6] was used in this investigation to assess how pro-inflammatory diets affect the risk of T2DM. It is the first systematic exploration of this association in a rural Chinese population. Existing evidence remains inconclusive, with a cross-sectional study of 5105 multi-ethnic Chinese adults reporting significantly elevated T2DM risk (OR = 3.27, 95% CI: 2.38–4.50) in high DII scorers [10]. Similarly, another cross-sectional study from the Iranian RaNCD cohort reported that individuals on pro-inflammatory diets were 1.61 times more likely to develop T2DM than those following anti-inflammatory diets [47]. Other studies, however, report no significant association between DII and T2DM risk after adjusting for confounding factors. A Melbourne cohort study (*n* = 39,185), for example, showed a null association after adjusting for birth region [48]. Tison et al.’s cohort study of 8750 U.S. black/white adults found no association after adjustment for demographics and lifestyle factors [49]. Consistent with the Melbourne [48] and U.S. [49] cohort study findings, among the total participants, our results revealed a 1.2-fold increased T2DM risk in Q4 versus Q1 in unadjusted models, and a 7% increase in T2DM risk for every 1-SD increase in DII score, which attenuated and became non-significant after comprehensive multivariable adjustment. The differences in findings across studies may stem from factors such as sample size, dietary measurement variability, and study population heterogeneity. Cultural and migration-related dietary effects in the Melbourne study, for example, and ethnic differences between Chinese and Western diets, may explain some inconsistencies.

The non-significant DII-T2DM association among overall study participants may stem from three key factors. First, the homogeneous rural Henan population exhibits limited dietary diversity and sociocultural variation, which may reduce individual DII variability. This study found that T2DM patients had lower intakes of anti-inflammatory nutrients than non-T2DM individuals, consistent with other studies [50,51]. Analysis indicated that participants’ dietary intake of anti-inflammatory components fell below global averages, resulting in positive DII scores for each anti-inflammatory nutrient. This likely contributed to the generally higher DII scores observed in the present study. The scores ranged from −3.86 to +4.33, with a median of 2.58. This median value is slightly above those reported in other studies, including a Chinese cross-sectional study (mean DII: 0.81) [10] and a Melbourne cohort study (median DII: −1.0) [48]. The overall high levels of DII across our cohort may have reduced the statistical power to identify significant exposure-outcome relationships. Second, this study may have underestimated the T2DM incidence density in the population. The 5.1-year follow-up yielded a T2DM incidence density of 26.83/1000 person-years, lower than the China Kadoorie Biobank’s 9.2-year rural data follow-up (31.2/1000 person-years) [52]. This discrepancy may be related to the different follow-up durations, given that the clinical diagnosis of T2DM is a long pathological process. Finally, single baseline dietary assessments failed to capture dynamic DII changes over time, potentially diluting DII-T2DM associations within the short observation period if pro-inflammatory diets became more prevalent during the follow-up. Further research is therefore needed to clarify this issue.

Notably, females in the highest DII quartile had a 1.36-fold higher T2DM risk, with each 1-SD DII increase associated with a 16% risk elevation. In contrast, no association was observed in males. These sex-specific findings align with our a priori hypothesis articulated in the Introduction and are consistent with previous epidemiological observations. Farhangi and colleagues reported gender-specific variations in the DII–hyperglycemia association, with females showing a positive association (OR = 1.18, 95% CI:1.00–1.40) while males showed no significant effect [53]. Similarly, Kouvari et al. suggested that anti-inflammatory diets may be more effective in preventing diabetes in women [16]. More recently, Zhang et al. reported similar sex-specific patterns in US women [18], corroborating our findings from a Chinese rural population. The mechanisms behind these gender differences are unknown, but research indicates that women may be more sensitive to inflammation-induced insulin resistance [54], while estrogen serves as a pivotal regulator in inflammation and metabolism [55], possibly making postmenopausal women more sensitive to pro-inflammatory dietary components and thus increasing T2DM risk [56]. To address the potential influence of menopausal status and pregnancies on the observed association in our study population, we conducted a female-specific sensitivity analysis with additional adjustment for these factors in Model 3. The results remained robust, suggesting that the DII-T2DM association observed in women in our cohort appears to be largely independent of menopausal status and pregnancies. Nevertheless, several methodological considerations should be acknowledged. First, there may be residual confounding by other sex-specific factors (such as physical activity intensity). Second, while our validated FFQ minimizes measurement error, differential bias by sex remains a theoretical possibility. Future research with more detailed hormonal measurements and longitudinal assessment of menopausal transition would further elucidate the biological mechanisms underlying these sex-specific associations.

This study identified synergistic interactions between central obesity metrics (WC and WHR) and DII in T2DM risk, with the synergistic modifying effect between abnormal WC/WHR and high DII contributing approximately 22% and 31% to total T2DM risk, respectively. No such effects were observed for BMI or WHtR. Further, sex-stratified analyses revealed distinct patterns of interaction. Among males, significant multiplicative interactions were observed between high DII and multiple obesity metrics, including BMI (HR: 2.12, *p* = 0.019), WHR (HR: 2.14, *p* = 0.027), and WC (HR: 2.41, *p* = 0.003), with WC also showing significant additive interaction (RERI = 0.83; AP = 0.41). In contrast, females showed no significant multiplicative or additive interactions between high DII and any obesity metric, although significant joint associations were observed for WHR- and WC-defined obesity with high DII. The lack of interaction with WHtR across all analyses is likely due to its weaker correlation with visceral adiposity compared to WC and WHR. A recent study based on clinically obtained imaging-measured fat distribution data (*n* = 1497), and using the UK Biobank prospective cohort (*n* = 322,023) for validation, demonstrated that WHR showed the strongest correlation with central fat, followed by moderate correlation for WC, while WHtR and BMI exhibited weak and fluctuating correlations [57]. Moreover, WHR outperformed other metrics as a predictor of T2DM and showed stronger associations with β-cell function [57]. These characteristics may explain WHR’s superior ability to capture synergistic effects with dietary inflammation in our study, whereas WHtR showed no such interaction. Regarding DII–obesity interactions in relation to diabetes risk, evidence is limited. Our finding in total participants aligns with that of Shu et al. [58], who reported no modifying effect of BMI on the DII–prediabetes association (*p* for interaction = 0.904). Our finding of significant DII interactions with central obesity, but not BMI, suggests that central adiposity may more effectively amplify dietary inflammation’s effects on T2DM development. Regarding gender-specific patterns, our findings in men contradict Guinter et al.’s findings from their male US cohort [23], which reported no obesity effect modification on the DII-T2DM association. In female participants, a cohort study involving 70,991 women in France identified a significant multiplicative interaction between DII and BMI in modulating the risk of T2DM (*p* < 0.001) [17]. In contrast, no such interaction was observed between pro-inflammatory diets and obesity in a cohort study of 3849 Hispanic women (*p* = 0.730) [22]. Our findings are highly consistent with the latter. These inconsistencies across studies may be attributed to core differences in dietary patterns and obesity phenotypes between Chinese and Western populations, as well as broader variations in ethnic and lifestyle contexts, which merit further investigation.

Mechanistically, central obesity, especially visceral fat accumulation, releases multiple inflammatory mediators (such as TNF-α, IL-6, CRP) [59] that impair insulin signaling mechanisms, leading to insulin resistance and T2DM development [60]. Visceral fat also more strongly associates with β-cell function decline than BMI [61]. When a high-inflammatory dietary pattern (high DII) coexists with central obesity, the two may act synergistically through common inflammatory pathways or the additive effect of inflammatory pathways to exacerbate insulin resistance and β-cell dysfunction [62]. The more pronounced interaction between a pro-inflammatory diet and obesity in its effects on T2DM risk in males compared to females may reflect sex-specific biological differences. Males tend to have greater visceral adipose tissue than females at comparable BMI [63], while androgens may promote adipogenesis and pro-inflammatory macrophage accumulation [15]. Conversely, premenopausal estrogen in females exerts anti-inflammatory effects and regulates insulin signaling in adipose tissue [64], potentially buffering the synergistic amplification between dietary inflammation and obesity. Additionally, females have proportionally more subcutaneous than visceral fat [65], with subcutaneous fat exhibiting lower metabolic activity and inflammatory properties; therefore, while females with both high DII and central obesity remain at elevated risk, the underlying mechanism may involve independent additive effects rather than synergistic amplification. Our findings highlight that not all central obesity metrics equally capture the metabolic phenotype most susceptible to dietary inflammatory insults. T2DM prevention should jointly target dietary inflammation and central obesity, prioritizing individuals with elevated WHR/WC. Centrally obese individuals may gain greater benefit from anti-inflammatory diets. Moreover, T2DM prevention strategies should be gender-tailored for precision prevention. The limited sample size of our gender-stratified analyses warrants further validation of the gender effect on this interaction in larger cohorts.

Notably, no significant multiplicative or additive interactions were observed between DII and any dyslipidemia subtype (high TC, TG, LDL-C, or low HDL-C) in either total participants or sex-stratified analyses. This null finding requires careful interpretation. Classifying dyslipidemia into subtypes resulted in limited sample sizes per category, which may have reduced the statistical power to detect subtle interactions. To our knowledge, no previous study has directly examined interactions between DII and dyslipidemia subtypes on T2DM risk, although the associations between DII and these metabolic factors have been established. Multiple studies have demonstrated that DII is significantly associated with dyslipidemia [25] and various lipid parameters [66], and that higher DII scores correlate with elevated triglycerides and adverse lipid profiles among T2DM patients [47,67]; however, these studies focused on direct associations rather than testing for statistical interactions. Mechanistically, chronic low-grade inflammation serves as a common pathophysiological basis for both T2DM and dyslipidemia [68], while the bidirectional relationship between glucose and lipid metabolism is well-established [47,69]. Pro-inflammatory diets can affect both lipid metabolism [66] and glucose homeostasis [68] through systemic inflammation and metabolic signaling. Despite these mechanistic connections suggesting potential synergy, our findings indicate that DII and dyslipidemia may exert largely independent effects on T2DM risk at the population level. This pattern differs from the interactions observed between DII and central obesity (WHR/WC), possibly because diet-induced inflammation and lipid abnormalities operate through partially distinct pathways—dietary inflammation primarily affecting systemic inflammatory status and insulin sensitivity, while dyslipidemia reflects multifactorial metabolic dysregulation [70] involving genetic, hormonal, and lifestyle factors beyond inflammation alone. This multifactorial basis may dilute or confound the detection of a specific, strong synergistic interaction with dietary inflammation alone for T2DM risk. Future studies with larger sample sizes, comprehensive dyslipidemia assessments, and repeated measurements are needed to confirm these findings across diverse populations.

The primary strengths of this study lie in its prospective cohort design and the application of both multiplicative and additive interaction models, enabling a thorough assessment of how DII interacts with various obesity metrics and dyslipidemia types in influencing T2DM risk. Nevertheless, the present study has several limitations. First, dietary data were derived from self-reported FFQ, which may introduce recall bias. Second, due to data limitations and population dietary characteristics, we utilized 24 out of 45 food parameters for DII calculations, which may have affected the assessment and comparability with studies using the full 45-parameter DII. Specifically, the reduced parameter set may lead to different absolute DII score ranges compared to studies using more comprehensive assessments, potentially limiting direct numerical comparisons across populations. Shivappa et al., however, found that DII calculations with 17, 28, and 44 food parameters were all positively associated with relevant inflammatory factors [71,72], showing similar effect sizes, and suggesting that the core inflammatory assessment capacity is preserved despite using fewer parameters. This supports the validity of our approach for examining DII-T2DM associations within our cohort, though caution should be exercised when comparing absolute DII values with other studies. Third, despite our comprehensive adjustment for multiple potential confounding factors, the potential influence of residual confounding cannot be entirely eliminated. Fourth, as the sample originated from a rural Chinese population, the generalizability of our findings to other ethnic or urban groups is limited; therefore, future research should validate these findings in more diverse populations. Fifth, as data on specific inflammatory diseases (such as rheumatoid arthritis and inflammatory bowel disease) were not collected, we could not account for them in the analysis; as a result, there may be residual confounding. Sixth, this study relied on a single baseline FFQ without repeated dietary measurements, which may fail to capture temporal changes in dietary patterns over the follow-up period, potentially leading to exposure misclassification and regression dilution bias that could attenuate observed associations [73]. Future studies should incorporate dynamic dietary assessments to better investigate the causal relationship linking DII to T2DM.

## 5. Conclusions

This study identified gender differences in the association between DII and T2DM risk, with significant associations observed in Chinese women. Additionally, positive multiplicative and additive interactions were observed between DII and central obesity metrics (WC and WHR), indicating that pro-inflammatory diets and central obesity may synergistically elevate T2DM risk through a common inflammatory pathway. Future research should enroll larger, demographically diverse cohorts to validate these findings and investigate underlying interaction mechanisms.

## Figures and Tables

**Table 1:** Baseline characteristics of the study participants by T2DM status.

Variables	Total (*n* = 8055)	T2DM (*n* = 1034)	Non-T2DM (*n* = 7021)	*p* Value
Age, years	56.00 (47.00–65.00)	59.00 (50.00–66.00)	56.00 (47.00–65.00)	<0.001
Male (%)	3241 (40.24)	404 (39.07)	2837 (40.41)	0.414
High school and above (%)	801 (9.96)	84 (8.12)	717 (10.23)	0.035
Married/cohabiting (%)	7278 (90.42)	918 (88.95)	6360 (90.64)	0.086
Mean individual monthly income ≤1000 CNY (%)	7342 (91.99)	948 (92.22)	6394 (91.96)	0.961
Current smoking (%)	1595 (19.80)	198 (19.15)	1397 (19.90)	0.463
Alcohol drinking (%)	973 (12.08)	121 (11.70)	852 (12.14)	0.690
Sleep duration, h/day	8.00 (7.00, 9.00)	8.00 (7.00, 9.50)	8.00 (7.00, 9.00)	0.903
Ideal PA (%)	6313 (78.37)	822 (79.50)	5491 (78.21)	0.347
DII score	2.58 (1.78, 2.92)	2.63 (1.79, 2.95)	2.57 (1.78, 2.91)	0.131
BMI, kg/m^2^	24.70 (22.31, 27.20)	26.32 (24.06, 28.69)	24.42 (22.10, 26.89)	<0.001
WC, cm	84.25 (77.25, 91.25)	89.50 (82.75, 96.00)	83.50 (77.00, 90.25)	<0.001
WHtR	0.53 (0.49, 0.58)	0.56 (0.52, 0.60)	0.53 (0.48, 0.57)	<0.001
WHR	0.89 (0.85, 0.94)	0.93 (0.88, 0.97)	0.89 (0.84, 0.94)	<0.001
FPG, mmol/L	5.10 (4.70, 5.50)	5.54 (5.10, 6.02)	5.05 (4.66, 5.42)	<0.001
SBP, mmHg	123.67 (112.00, 138.33)	129.00 (116.67, 142.33)	123.00 (111.33, 137.67)	<0.001
DBP, mmHg	77.00 (70.00, 85.00)	79.67 (72.33, 87.00)	76.67 (69.67, 84.67)	<0.001
TC, mmol/L	4.34 (3.77, 4.97)	4.56 (3.98, 5.20)	4.31 (3.75, 4.94)	<0.001
TG, mmol/L	1.36 (0.97, 1.97)	1.68 (1.20, 2.36)	1.32 (0.94, 1.90)	<0.001
HDL-C, mmol/L	1.08 (0.93, 1.25)	1.04 (0.90, 1.21)	1.09 (0.93, 1.26)	<0.001
LDL-C, mmol/L	2.52 (2.06, 3.05)	2.62 (2.16, 3.21)	2.50 (2.04, 3.02)	<0.001
Family history of diabetes (%)	896 (11.12)	177 (17.12)	719 (10.24)	<0.001
Hypertension (%)	2910 (36.13)	480 (46.42)	2430 (34.61)	<0.001
Dyslipidemia (%)	3742 (52.11)	580 (62.57)	3162 (50.56)	<0.001

Abbreviations: T2DM, type 2 diabetes mellitus; PA, physical activity; DII, dietary inflammatory index; BMI, body mass index; WC, waist circumference; WHtR, waist-to-height ratio; WHR, waist-to-hip ratio; FPG, fasting plasma glucose; SBP, systolic blood pressure; DBP, diastolic blood pressure; TC, total cholesterol; TG, triglycerides; HDL-C, high-density lipoprotein cholesterol; LDL-C, low-density lipoprotein cholesterol. Note: Variables are presented as the median (interquartile range) or frequency (percentage).

**Table 2:** HR (95% CI) of incident T2DM for study participants in different DII quartile groups by gender.

Quartile Group of DII		Cases	Person-Years	Incidence Density *	Model 1		Model 2		Model 3	
					HR (95% CI)	*p* Value	HR (95% CI)	*p* Value	HR (95% CI)	*p* Value
Total										
	Q1	256	9774.77	26.19	1		1		1	
	Q2	234	9672.66	24.19	1.03 (0.86, 1.23)	0.751	1.00 (0.84, 1.20)	0.993	1.08 (0.89, 1.32)	0.440
	Q3	267	9562.37	27.92	1.19 (1.00, 1.42)	0.045	1.09 (0.92, 1.30)	0.318	1.13 (0.91, 1.41)	0.277
	Q4	277	9523.66	29.09	1.20 (1.01, 1.42)	0.038	1.05 (0.89, 1.26)	0.555	1.15 (0.93, 1.43)	0.198
	*P* trend				0.036		0.454		0.193	
	Per 1-SD increase				1.07 (1.01, 1.13)	0.030	1.02 (0.96, 1.09)	0.504	1.06 (0.98, 1.15)	0.151
Male										
	Q1	141	4791.80	29.43	1		1		1	
	Q2	104	4101.89	25.35	1.00 (0.77, 1.29)	0.980	0.96 (0.74, 1.24)	0.740	0.96 (0.71, 1.29)	0.779
	Q3	88	3241.45	27.15	1.11 (0.85, 1.45)	0.450	1.03 (0.78, 1.34)	0.841	1.02 (0.72, 1.43)	0.934
	Q4	71	3079.70	23.05	0.88 (0.66, 1.18)	0.393	0.81 (0.59, 1.07)	0.124	0.82 (0.57, 1.18)	0.285
	*P* trend				0.796		0.319		0.494	
	per 1-SD increase				0.98 (0.90, 1.07)	0.645	0.95 (0.87, 1.03)	0.200	0.94 (0.83, 1.07)	0.361
Female										
	Q1	115	4982.97	23.08	1		1		1	
	Q2	130	5570.76	23.34	1.10 (0.85, 1.41)	0.476	1.07 (0.83, 1.37)	0.619	1.19 (0.90, 1.57)	0.227
	Q3	179	6320.92	28.32	1.33 (1.06, 1.69)	0.016	1.20 (0.95, 1.52)	0.138	1.22 (0.91, 1.63)	0.191
	Q4	206	6443.97	31.97	1.48 (1.17, 1.85)	0.001	1.25 (0.99, 1.57)	0.064	1.36 (1.03, 1.81)	0.031
	*P* trend				0.003		0.086		0.056	
	per 1-SD increase				1.17 (1.08, 1.27)	<0.001	1.10 (1.01, 1.20)	0.030	1.16 (1.04, 1.29)	0.008

Abbreviations: T2DM, type 2 diabetes mellitus; DII, dietary inflammatory index; HR, hazard ratio; CI, confidence interval; SD, standard deviation. Model 1: Unadjusted. Model 2: Adjusted for age, gender, educational level, marital status, and average monthly income. Model 3: Adjusted for model 2 variables and smoking status, drinking status, physical activity, sleep duration, BMI status, family history of diabetes, energy intake, hypertension, dyslipidemia, and fasting plasma glucose. Note: For single-gender dataset analyses, gender was not included as a covariate. DII range: Q1 (<1.78), Q2 (1.78~2.58), Q3 (2.58~2.92), Q4 (≥2.92); DII per 1-SD = 1.10 point. * Per 1000 person-years.

**Table 3:** Interactions and joint associations of obesity metrics and binary DII groups on the risk of T2DM in total participants.

Obesity Groups	DII Groups	Joint Association		Multiplicative Interaction		Additive Interaction	
		HR (95% CI)	*p* Value	HR (95% CI)	*p* Value	RERI (95% CI)	AP (95% CI)
BMI							
Normal	Low	1					
Normal	High	0.98 (0.81, 1.18)	0.802				
Obesity	Low	0.68 (0.52, 0.89)	0.005				
Obesity	High	0.78 (0.56, 1.07)	0.125	1.17 (0.86, 1.61)	0.315	0.12 (−0.15, 0.39)	0.16 (−0.27, 0.43)
WHtR							
Normal	Low	1					
Normal	High	0.91 (0.62, 1.32)	0.608				
Obesity	Low	1.40 (1.11, 1.76)	0.005				
Obesity	High	1.46 (1.13, 1.90)	0.004	1.16 (0.77, 1.73)	0.483	0.16 (−0.33, 0.52)	0.11 (−0.22, 0.36)
WHR							
Normal	Low	1					
Normal	High	0.69 (0.47, 1.00)	0.053				
Obesity	Low	1.34 (1.10, 1.65)	0.004				
Obesity	High	1.50 (1.19, 1.90)	0.001	1.62 (1.08, 2.44)	0.020	0.47 (0.07, 0.80)	0.31 (0.05, 0.52)
WC							
Normal	Low	1					
Normal	High	0.83 (0.64, 1.07)	0.144				
Obesity	Low	1.60 (1.31, 1.95)	<0.001				
Obesity	High	1.84 (1.45, 2.33)	<0.001	1.39 (1.02, 1.90)	0.038	0.41 (0.02, 0.81)	0.22 (0.00, 0.39)

Abbreviations: T2DM, type 2 diabetes mellitus; DII, dietary inflammatory index; HR, hazard ratio; CI, confidence interval; BMI, body mass index; WC, waist circumference; WHtR, waist-to-height ratio; WHR, waist-to-hip ratio; RERI, relative excess risk due to interaction; AP, attributable proportion due to interaction. Adjusted for: age, gender, educational level, marital status, average monthly income, smoking status, drinking status, physical activity, sleep duration, BMI status, family history of diabetes, energy intake, hypertension, dyslipidemia, and fasting plasma glucose. Note: The reference category for joint-association analysis is participants with normal BMI/WHR/WC/WHtR and a low DII score. Interpretation of additive interaction indices: RERI quantifies the excess disease risk attributable specifically to the interaction between two exposures (such as high DII and obesity), beyond the sum of their individual effects. AP estimates the proportion of disease risk in the doubly exposed group that is due to this interaction. An additive interaction is suggested when the 95% confidence interval for both RERI and AP does not include 0.

**Table 4:** Interactions and joint associations of dyslipidemia types and binary DII groups on the risk of T2DM in total participants.

Dyslipidemia Groups	DII Groups	Joint Association		Multiplicative Interaction		Additive Interaction	
		HR (95% CI)	*p* Value	HR (95% CI)	*p* Value	RERI (95% CI)	AP (95% CI)
High TC							
No	Low	1					
No	High	1.01 (0.86, 1.19)	0.908				
Yes	Low	0.91 (0.65, 1.29)	0.613				
Yes	High	1.18 (0.70, 1.99)	0.551	1.28 (0.68, 2.39)	0.447	0.25 (−0.36, 1.09)	0.22 (−0.66, 0.46)
High TG							
No	Low	1					
No	High	1.03 (0.86, 1.24)	0.721				
Yes	Low	1.20 (0.99, 1.45)	0.061				
Yes	High	1.21 (0.92, 1.59)	0.185	0.97 (0.70, 1.35)	0.864	−0.03 (−0.41, 0.37)	−0.02 (−0.44, 0.22)
Low HDL-C							
No	Low	1					
No	High	1.15 (0.93, 1.41)	0.193				
Yes	Low	1.04 (0.84, 1.29)	0.718				
Yes	High	0.94 (0.72, 1.23)	0.671	0.79 (0.59, 1.06)	0.116	−0.25 (−0.59, 0.06)	−0.26 (−0.71, 0.03)
High LDL-C							
No	Low	1					
No	High	1.01 (0.86, 1.20)	0.864				
Yes	Low	0.60 (0.36, 0.99)	0.046				
Yes	High	0.81 (0.44, 1.48)	0.505	1.33 (0.60, 2.93)	0.477	0.20 (−0.36, 0.91)	0.24 (−1.05, 0.60)

Abbreviations: T2DM, type 2 diabetes mellitus; DII, dietary inflammatory index; HR, hazard ratio; CI, confidence interval; High TC, hypercholesterolemia; High TG, hypertriglyceridemia; Low HDL-C, low high-density lipoprotein cholesterol; High LDL-C, high low-density lipoprotein cholesterol; RERI, relative excess risk due to interaction; AP, attributable proportion due to interaction. Adjusted for: age, gender, educational level, marital status, average monthly income, smoking status, drinking status, physical activity, sleep duration, BMI status, family history of diabetes, energy intake, hypertension, dyslipidemia, and fasting plasma glucose. Note: The reference category for joint-association analysis is participants without the specific dyslipidemia and a low DII score. Interpretation of additive interaction indices: RERI quantifies the excess disease risk attributable specifically to the interaction between two exposures (such as high DII and high TC), beyond the sum of their individual effects. AP estimates the proportion of disease risk in the doubly exposed group that is due to this interaction. An additive interaction is suggested when the 95% confidence interval for both RERI and AP does not include 0.

## Data Availability

Data are available from the corresponding author upon reasonable request.

## Supplementary Materials

The following supporting information can be downloaded at: https://www.mdpi.com/article/10.3390/nu18050738/s1, Figure S1: Flowchart of participants’ selection; Table S1: The calculation steps for the DII of study participants; Table S2: Analysis of multicollinearity among covariates; Table S3: Baseline characteristics of the study participants grouped by DII quartiles; Table S4: Baseline characteristics of the study participants by gender; Table S5: Dietary intake of each food parameter grouped by T2DM status; Table S6: Food parameter-specific DII scores grouped by overall DII quartiles among participants; Table S7: Interactions and joint associations of obesity metrics and binary DII groups on the risk of T2DM among males; Table S8: Interactions and joint associations of dyslipidemia types and binary DII groups on the risk of T2DM among males; Table S9: Interactions and joint associations of obesity metrics and binary DII groups on the risk of T2DM among females; Table S10: Interactions and joint associations of dyslipidemia types and binary DII groups on the risk of T2DM among females; Table S11: Subgroup analysis of the association between DII level (category and per 1-SD) and risk of T2DM; Table S12: Sensitivity analysis of the association between DII levels and T2DM: Exclusion of individuals who developed T2DM within the first two years of follow-up; Table S13: Sensitivity analysis of the association between DII levels and T2DM: Imputing missing values in variables using multiple imputation; Table S14: Sensitivity analysis of the association between DII levels and T2DM: Substituting BMI with alternative adiposity metrics (WHtR, WHR, or WC) in Model 3; Table S15: Sensitivity analysis of the association between DII levels and T2DM in female participants: Further adjusted for covariates of menopausal status and pregnancies.

## References

[1] International Diabetes Federation IDF Diabetes Atlas 11th Edition. https://diabetesatlas.org/resources/idf-diabetes-atlas-2025/.

[2] Pouvreau C., Dayre A., Butkowski E.G., de Jong B., Jelinek H.F. (2018). Inflammation and oxidative stress markers in diabetes and hypertension. J. Inflamm. Res..

[3] Brahimaj A., Ligthart S., Ghanbari M., Ikram M.A., Hofman A., Franco O.H., Kavousi M., Dehghan A. (2017). Novel inflammatory markers for incident pre-diabetes and type 2 diabetes: The Rotterdam Study. Eur. J. Epidemiol..

[4] Esposito K., Marfella R., Ciotola M., Di Palo C., Giugliano F., Giugliano G., D’Armiento M., D’Andrea F., Giugliano D. (2004). Effect of a mediterranean-style diet on endothelial dysfunction and markers of vascular inflammation in the metabolic syndrome: A randomized trial. JAMA.

[5] Christ A., Lauterbach M., Latz E. (2019). Western Diet and the Immune System: An Inflammatory Connection. Immunity.

[6] Shivappa N., Steck S.E., Hurley T.G., Hussey J.R., Hébert J.R. (2014). Designing and developing a literature-derived, population-based dietary inflammatory index. Public Health Nutr..

[7] Kotemori A., Sawada N., Iwasaki M., Yamaji T., Shivappa N., Hebert J.R., Ishihara J., Inoue M., Tsugane S. (2020). Validating the dietary inflammatory index using inflammatory biomarkers in a Japanese population: A cross-sectional study of the JPHC-FFQ validation study. Nutrition.

[8] Wirth M.D., Shivappa N., Davis L., Hurley T.G., Ortaglia A., Drayton R., Blair S.N., Hébert J.R. (2017). Construct Validation of the Dietary Inflammatory Index among African Americans. J. Nutr. Health Aging.

[9] Shen W., Cai L., Wang B., Li J., Sun Y., Chen Y., Xia F., Wang N., Lu Y. (2024). Associations of a proinflammatory diet, habitual salt intake, and the onset of type 2 diabetes: A prospective cohort study from the UK Biobank. Diabetes Obes. Metab..

[10] Fu W., Pei H., Shivappa N., Hebert J.R., Luo T., Tian T., Alimu D., Zhang Z., Dai J. (2021). Association between Dietary Inflammatory Index and Type 2 diabetes mellitus in Xinjiang Uyghur autonomous region, China. PeerJ.

[11] Motamedi A., Askari M., Mozaffari H., Homayounfrar R., Nikparast A., Ghazi M.L., Nejad M.M., Alizadeh S. (2022). Dietary Inflammatory Index in relation to Type 2 Diabetes: A Meta-Analysis. Int. J. Clin. Pract..

[12] Mauvais-Jarvis F., Manson J.E., Stevenson J.C., Fonseca V.A. (2017). Menopausal Hormone Therapy and Type 2 Diabetes Prevention: Evidence, Mechanisms, and Clinical Implications. Endocr. Rev..

[13] Tramunt B., Smati S., Grandgeorge N., Lenfant F., Arnal J.F., Montagner A., Gourdy P. (2020). Sex differences in metabolic regulation and diabetes susceptibility. Diabetologia.

[14] Ding E.L., Song Y., Malik V.S., Liu S. (2006). Sex differences of endogenous sex hormones and risk of type 2 diabetes: A systematic review and meta-analysis. JAMA.

[15] Varghese M., Griffin C., Singer K. (2017). The Role of Sex and Sex Hormones in Regulating Obesity-Induced Inflammation. Adv. Exp. Med. Biol..

[16] Kouvari M., Panagiotakos D.B., Naumovski N., Chrysohoou C., Georgousopoulou E.N., Yannakoulia M., Tousoulis D., Pitsavos C. (2020). Dietary anti-inflammatory index, metabolic syndrome and transition in metabolic status; a gender-specific analysis of ATTICA prospective study. Diabetes Res. Clin. Pract..

[17] Laouali N., Mancini F.R., Hajji-Louati M., El Fatouhi D., Balkau B., Boutron-Ruault M.C., Bonnet F., Fagherazzi G. (2019). Dietary inflammatory index and type 2 diabetes risk in a prospective cohort of 70,991 women followed for 20 years: The mediating role of BMI. Diabetologia.

[18] Mo T., Wei M., Fu J. (2024). Dietary inflammatory index and type 2 diabetes in US women: A cross-sectional analysis of the National Health and Nutrition Examination Survey, 2007–2018. Front. Nutr..

[19] Org E., Mehrabian M., Parks B.W., Shipkova P., Liu X., Drake T.A., Lusis A.J. (2016). Sex differences and hormonal effects on gut microbiota composition in mice. Gut Microbes.

[20] Hariharan R., Odjidja E.N., Scott D., Shivappa N., Hébert J.R., Hodge A., de Courten B. (2022). The dietary inflammatory index, obesity, type 2 diabetes, and cardiovascular risk factors and diseases. Obes. Rev..

[21] Kawai T., Autieri M.V., Scalia R. (2021). Adipose tissue inflammation and metabolic dysfunction in obesity. Am. J. Physiol. Cell Physiol..

[22] Zuercher M.D., Harvey D.J., Au L.E., Shadyab A.H., Santiago-Torres M., Liu S., Shivappa N., Hébert J.R., Robbins J.A., Garcia L. (2024). Energy-Adjusted Dietary Inflammatory Index and Diabetes Risk in Postmenopausal Hispanic Women. J. Acad. Nutr. Diet..

[23] Guinter M.A., Merchant A.T., Tabung F.K., Wirth M.D., Shivappa N., Hurley T.G., Hebert J.R., Sui X., Blair S.N., Steck S.E. (2019). Adiposity does not modify the effect of the dietary inflammatory potential on type 2 diabetes incidence among a prospective cohort of men. J. Nutr. Intermed. Metab..

[24] Hong N., Lin Y., Ye Z., Yang C., Huang Y., Duan Q., Xie S. (2022). The relationship between dyslipidemia and inflammation among adults in east coast China: A cross-sectional study. Front. Immunol..

[25] Chen X., Hou C., Yao L., Li J., Gui M., Wang M., Zhou X., Lu B., Fu D. (2023). Dietary inflammation index is associated with dyslipidemia: Evidence from national health and nutrition examination survey, 1999–2019. Lipids Health Dis..

[26] Zhang M., Zhao Y., Sun L., Xi Y., Zhang W., Lu J., Hu F., Shi X., Hu D. (2021). Cohort Profile: The Rural Chinese Cohort Study. Int. J. Epidemiol..

[27] Yang Y. (2018). China Food Composition Tables, Standard Edition, 6th Revision.

[28] Ren Z., Zhao A., Wang Y., Meng L., Szeto I.M., Li T., Gong H., Tian Z., Zhang Y., Wang P. (2018). Association between Dietary Inflammatory Index, C-Reactive Protein and Metabolic Syndrome: A Cross-Sectional Study. Nutrients.

[29] Zhao Y., Sun H., Zhang W., Xi Y., Shi X., Yang Y., Lu J., Zhang M., Sun L., Hu D. (2021). Elevated triglyceride-glucose index predicts risk of incident ischaemic stroke: The Rural Chinese cohort study. Diabetes Metab..

[30] Hassani S., Ovbiagele B., Markovic D., Towfighi A. (2024). Association Between Abnormal Sleep Duration and Stroke in the United States. Neurology.

[31] Craig C.L., Marshall A.L., Sjöström M., Bauman A.E., Booth M.L., Ainsworth B.E., Pratt M., Ekelund U., Yngve A., Sallis J.F. (2003). International physical activity questionnaire: 12-country reliability and validity. Med. Sci. Sports Exerc..

[32] Ainsworth B.E., Haskell W.L., Herrmann S.D., Meckes N., Bassett D.R., Tudor-Locke C., Greer J.L., Vezina J., Whitt-Glover M.C., Leon A.S. (2011). 2011 Compendium of Physical Activities: A second update of codes and MET values. Med. Sci. Sports Exerc..

[33] Piercy K.L., Troiano R.P., Ballard R.M., Carlson S.A., Fulton J.E., Galuska D.A., George S.M., Olson R.D. (2018). The Physical Activity Guidelines for Americans. JAMA.

[34] Chobanian A.V., Bakris G.L., Black H.R., Cushman W.C., Green L.A., Izzo J.L., Jones D.W., Materson B.J., Oparil S., Wright J.T. (2003). Seventh report of the joint national committee on prevention, detection, evaluation, and treatment of high blood pressure. Hypertension.

[35] Bairaktari E., Hatzidimou K., Tzallas C., Vini M., Katsaraki A., Tselepis A., Elisaf M., Tsolas O. (2000). Estimation of LDL cholesterol based on the Friedewald formula and on apo B levels. Clin. Biochem..

[36] National Health Commission of the People’s Republic of China Criteria of Weight for Adults: WS/T 428-2013. http://www.nhc.gov.cn/ewebeditor/uploadfile/2013/08/20130808135715967.pdf.

[37] World Health Organization (2011). Global Status Report on Noncommunicable Diseases 2010.

[38] Hsieh S.D., Muto T. (2005). The superiority of waist-to-height ratio as an anthropometric index to evaluate clustering of coronary risk factors among non-obese men and women. Prev. Med..

[39] Ren Z. (2007). Guidelines on prevention and treatment of blood lipid abnormality in Chinese adults. Chin. J. Cardiol..

[40] Jia W., Weng J., Zhu D., Ji L., Lu J., Zhou Z., Zou D., Guo L., Ji Q., Chen L. (2019). Standards of medical care for type 2 diabetes in China 2019. Diabetes Metab. Res. Rev..

[41] Knol M.J., VanderWeele T.J. (2012). Recommendations for presenting analyses of effect modification and interaction. Int. J. Epidemiol..

[42] Knol M.J., VanderWeele T.J., Groenwold R.H., Klungel O.H., Rovers M.M., Grobbee D.E. (2011). Estimating measures of interaction on an additive scale for preventive exposures. Eur. J. Epidemiol..

[43] Li R., Chambless L. (2007). Test for additive interaction in proportional hazards models. Ann. Epidemiol..

[44] Zhuo S., Zhang B., Zhang J., Yang M., Yu Z. (2023). Effects of dietary inflammatory index, blood lead levels, and flavonoid intake on stroke risk in older Americans: A cross-sectional study. J. Stroke Cerebrovasc. Dis..

[45] VanderWeele T.J., Knol M.J. (2014). A tutorial on interaction. Epidemiol. Methods.

[46] Brankovic M., Kardys I., Steyerberg E.W., Lemeshow S., Markovic M., Rizopoulos D., Boersma E. (2019). Understanding of interaction (subgroup) analysis in clinical trials. Eur. J. Clin. Investig..

[47] Namazi N., Anjom-Shoae J., Najafi F., Ayati M.H., Darbandi M., Pasdar Y. (2023). Pro-inflammatory diet, cardio-metabolic risk factors and risk of type 2 diabetes: A cross-sectional analysis using data from RaNCD cohort study. BMC Cardiovasc. Disord..

[48] Hodge A.M., Karim M.N., Hébert J.R., Shivappa N., de Courten B. (2021). Association between Diet Quality Indices and Incidence of Type 2 Diabetes in the Melbourne Collaborative Cohort Study. Nutrients.

[49] Tison S.E., Shikany J.M., Long D.L., Carson A.P., Cofield S.S., Pearson K.E., Howard G., Judd S.E. (2022). Differences in the Association of Select Dietary Measures with Risk of Incident Type 2 Diabetes. Diabetes Care.

[50] Cheloi N., Asgari Z., Ershadi S., Naseri R., Sharifi A. (2025). Comparison of Body Mass Index, Energy and Macronutrient Intake, and Dietary Inflammatory Index Between Type 2 Diabetic and Healthy Individuals. J. Res. Health Sci..

[51] Xu H., Luo J., Huang J., Wen Q. (2018). Flavonoids intake and risk of type 2 diabetes mellitus: A meta-analysis of prospective cohort studies. Medicine.

[52] Xu Y., Lu J., Li M., Wang T., Wang K., Cao Q., Ding Y., Xiang Y., Wang S., Yang Q. (2024). Diabetes in China part 1: Epidemiology and risk factors. Lancet Public Health.

[53] Farhangi M.A., Nikniaz L., Nikniaz Z., Dehghan P. (2020). Dietary inflammatory index potentially increases blood pressure and markers of glucose homeostasis among adults: Findings from an updated systematic review and meta-analysis. Public Health Nutr..

[54] Kautzky-Willer A., Harreiter J., Pacini G. (2016). Sex and Gender Differences in Risk, Pathophysiology and Complications of Type 2 Diabetes Mellitus. Endocr. Rev..

[55] Vegeto E., Benedusi V., Maggi A. (2008). Estrogen anti-inflammatory activity in brain: A therapeutic opportunity for menopause and neurodegenerative diseases. Front. Neuroendocrinol..

[56] Li Y., Guo X., Ge J., Li Q., Chen X., Zhu Y., Yuan H., Geng S., Liu Y. (2025). Sex differences in associations of metabolic inflammation and insulin resistance with incident type 2 diabetes mellitus: A retrospective cohort of adults with annual health examinations. Lipids Health Dis..

[57] Liu N., Wang B., Zhang G., Shen M., Cheng P., Guo Z., Zuo L., Yang J., Guo M., Wang M. (2025). Waist-to-hip ratio better reflect beta-cell function and predicts diabetes risk in adult with overweight or obesity. Ann. Med..

[58] Shu Y., Wu X., Wang J., Ma X., Li H., Xiang Y. (2022). Associations of Dietary Inflammatory Index with Prediabetes and Insulin Resistance. Front. Endocrinol..

[59] Makki K., Froguel P., Wolowczuk I. (2013). Adipose tissue in obesity-related inflammation and insulin resistance: Cells, cytokines, and chemokines. ISRN Inflamm..

[60] Hotamisligil G.S. (2017). Inflammation, metaflammation and immunometabolic disorders. Nature.

[61] Wagenknecht L.E., Langefeld C.D., Scherzinger A.L., Norris J.M., Haffner S.M., Saad M.F., Bergman R.N. (2003). Insulin sensitivity, insulin secretion, and abdominal fat: The Insulin Resistance Atherosclerosis Study (IRAS) Family Study. Diabetes.

[62] Gregor M.F., Hotamisligil G.S. (2011). Inflammatory mechanisms in obesity. Annu. Rev. Immunol..

[63] Lemieux S., Prud’homme D., Bouchard C., Tremblay A., Després J.P. (1993). Sex differences in the relation of visceral adipose tissue accumulation to total body fatness. Am. J. Clin. Nutr..

[64] Mauvais-Jarvis F., Clegg D.J., Hevener A.L. (2013). The role of estrogens in control of energy balance and glucose homeostasis. Endocr. Rev..

[65] Palmer B.F., Clegg D.J. (2015). The sexual dimorphism of obesity. Mol. Cell Endocrinol..

[66] Phillips C.M., Shivappa N., Hébert J.R., Perry I.J. (2018). Dietary Inflammatory Index and Biomarkers of Lipoprotein Metabolism, Inflammation and Glucose Homeostasis in Adults. Nutrients.

[67] Rahimlou M., Ahmadi A.R., Cheraghian B., Baghdadi G., Ghalishourani S.S., Nozarian S., Hashemi S.J., Rahimi Z., Jahromi N.B., Hosseini S.A. (2024). The association between dietary inflammatory index with some cardio-metabolic risk indices among the patients with type 2 diabetes from Hoveyzeh cohort study: A cross-sectional study. BMC Endocr. Disord..

[68] Xu J., Xie L., Fan R., Shi X., Xu W., Dong K., Ma D., Yan Y., Zhang S., Sun N. (2025). The role of dietary inflammatory index in metabolic diseases: The associations, mechanisms, and treatments. Eur. J. Clin. Nutr..

[69] Xiao C., Dash S., Morgantini C., Lewis G.F. (2013). Novel role of enteral monosaccharides in intestinal lipoprotein production in healthy humans. Arterioscler. Thromb. Vasc. Biol..

[70] Dakal T.C., Xiao F., Bhusal C.K., Sabapathy P.C., Segal R., Chen J., Bai X. (2025). Lipids dysregulation in diseases: Core concepts, targets and treatment strategies. Lipids Health Dis..

[71] Shivappa N., Hébert J.R., Rietzschel E.R., De Buyzere M.L., Langlois M., Debruyne E., Marcos A., Huybrechts I. (2015). Associations between dietary inflammatory index and inflammatory markers in the Asklepios Study. Br. J. Nutr..

[72] Shivappa N., Steck S.E., Hurley T.G., Hussey J.R., Ma Y., Ockene I.S., Tabung F., Hébert J.R. (2014). A population-based dietary inflammatory index predicts levels of C-reactive protein in the Seasonal Variation of Blood Cholesterol Study (SEASONS). Public Health Nutr..

[73] Hutcheon J.A., Chiolero A., Hanley J.A. (2010). Random measurement error and regression dilution bias. BMJ.

