# Supplementary material for: The Dietary Inflammatory Index and Incident Risk of Type 2 Diabetes Mellitus: Interactions with Obesity and Dyslipidemia in a Prospective Cohort Study
Source: Nutrients. 2026 Feb 25;18(5):738. doi: 10.3390/nu18050738 (PMC12986315; doi:10.3390/nu18050738)
Supplement: Supplementary file 1 [file nutrients-18-00738-s001.zip › nutrients-4107551-supplementary.pdf]

Supplementary Materials

Figure S1. Flowchart of participants' selection.

Table S1. The calculation steps for the DII of study participants.

Table S2. Analysis of multicollinearity among covariates.

Table S3. Baseline characteristics of the study participants grouped by DII quartiles.

Table S4. Baseline characteristics of the study participants by gender.

Table S5. Dietary intake of each food parameter grouped by T2DM status.

Table S6. Food parameter-specific DII scores grouped by overall DII quartiles among participants.

Table S7. Interactions and joint associations of obesity metrics and binary DII groups on the risk of T2DM among males.

Table S8. Interactions and joint associations of dyslipidemia types and binary DII groups on the risk of T2DM among males.

Table S9. Interactions and joint associations of obesity metrics and binary DII groups on the risk of T2DM among females.

Table S10. Interactions and joint associations of dyslipidemia types and binary DII groups on the risk of T2DM among females.

Table S11. Subgroup analysis of the association between DII level (category and per 1-SD) and risk of T2DM.

Table S12. Sensitivity analysis of the association between DII levels and T2DM: Exclusion of individuals who developed T2DM within the first two years of follow-up.

Table S13. Sensitivity analysis of the association between DII levels and T2DM: Imputing missing values in variables using multiple imputation.

Table S14. Sensitivity analysis of the association between DII levels and T2DM: Substituting BMI with alternative adiposity metrics (WHtR, WHR, or WC) in Model 3.

Table S15. Sensitivity analysis of the association between DII levels and T2DM in female participants: Further adjusted for covariates of menopausal status and pregnancies.

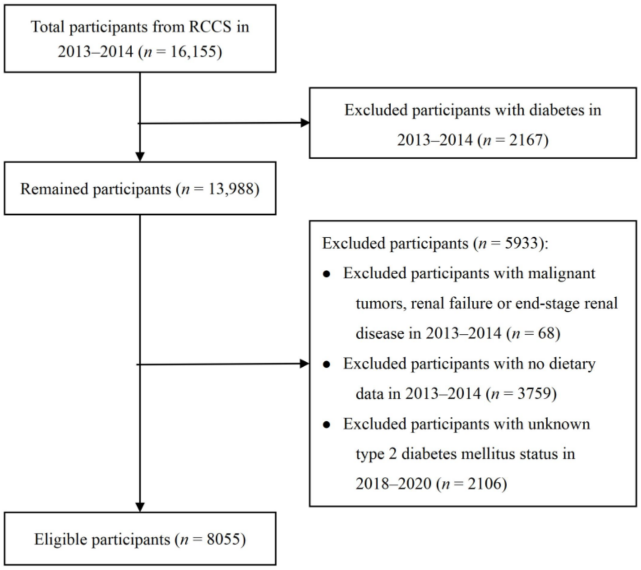

Figure S1. Flowchart of participants' selection.

Table S1. The calculation steps for the DII of study participants.

Step	Step description	Formula/Transformation Logic	Output
1	Dietary data conversion	Calculate daily food intake from FFQ and convert to 24* food parameters using the Chinese Food Composition Table.	Individual daily intake of each food parameter
2	Z-Score calculation (Standardization)	$Z = \frac{\chi_{i,j} - \mu_i}{\sigma_i}$ *j* = study participant; *i* = each food parameter; *x* = daily intake of each food parameter; μ = global daily mean intake; σ = standard deviation of the global daily intake	Standardized Z-score (dimensionless)
3	Convert Z-scores to percentile values & Symmetrization (To reduce outliers and the effect of right skewness)	1. Convert Z-score to percentile; 2. Symmetrize: $Symmetric\ Value = (Percentile_{i,j} \times 2) - 1$	Symmetric value (centered at 0, range for −1 to 1)
4	Calculate the food parameter-specific DII score	$DII_{i,j} = Symmetric\ Value_{i,j} \times e_i$ e_i = overall inflammatory effect score for each food parameter	Food parameter-specific DII score (reflecting contribution to inflammation)
5	Calculate the overall DII score	$DII_j = \sum_{i=1}^{n} DII_{i,j}$ *n* = 24 = total number of parameters	Overall DII (overall inflammatory potential of diet)

*: Energy (kcal), Carbohydrate (g), Protein (g), Total fat (g), Fiber (g), Cholesterol (mg), Saturated fat (g), monounsaturated fatty acids (MUFA) (g), polyunsaturated fatty acids (PUFA) (g), Niacin (mg), Thiamin (mg), Riboflavin (mg), Folic acid (μg), Vitamin A (μg), Vitamin C (mg), Vitamin E (mg), Fe (mg), Mg (mg), Se (μg), Zn (mg), β-Carotene (μg), Isoflavones (mg), Anthocyanidins (mg), and Alcohol (g).

Table S2. Analysis of multicollinearity among covariates.

Variables	VIF
Age	1.41
Gender	2.57
Educational level	1.06
Marital status	1.12
Average monthly income	1.02
Smoking status	2.17
Drinking status	1.29
Physical activity	1.14
Sleep duration	1.01
BMI status	1.11
Family history of diabetes	1.05
Energy intake	1.19
Hypertension	1.12
Dyslipidemia	1.06
Fasting plasma glucose	1.04

Note: VIF, Variance Inflation Factor; values <5.0 indicate acceptable multicollinearity.

Table S3. Baseline characteristics of the study participants grouped by DII quartiles.

Variables	Q1 (*n* = 2011)	Q2 (*n* = 2025)	Q3 (*n* = 2019)	Q4 (*n* = 2000)	*P* value
DII range	<1.78	1.78~2.58	2.58~2.92	≥2.92	
Age, years	53.00 (45.00, 62.00)	55.00 (46.00, 63.00)	57.00 (48.00, 66.00)	59.00 (50.00, 67.00)	<0.001
Male (%)	997 (49.58)	881 (43.51)	707 (35.02)	656 (32.80)	<0.001
High school and above (%)	262 (13.03)	234 (11.58)	192 (9.52)	113 (5.66)	<0.001
Married/cohabiting (%)	1891 (94.03)	1859 (91.85)	1771 (87.80)	1757 (87.98)	<0.001
Mean individual monthly income ≤1000 CNY (%)	1772 (88.69)	1833 (91.15)	1875 (93.52)	1880 (94.71)	<0.001
Current smoking (%)	510 (25.36)	450 (22.22)	345 (17.09)	290 (14.50)	<0.001
Alcohol drinking (%)	393 (19.54)	305 (15.06)	144 (7.13)	131 (6.55)	<0.001
Sleep duration, h/day	8.00 (7.00, 9.00)	8.00 (7.50, 9.00)	8.00 (7.00, 9.00)	8.00 (7.00, 10.00)	0.053
Ideal physical activity (%)	1561 (77.62)	1548 (76.44)	1621 (80.29)	1583 (79.15)	0.017
BMI, kg/m2	24.96 (22.49, 27.33)	24.65 (22.33, 27.10)	24.56 (22.10, 26.97)	24.56 (22.21, 27.24)	0.006
WC, cm	85.00 (78.00, 91.75)	84.25 (77.50, 91.00)	84.25 (77.00, 90.75)	84.25 (77.25, 91.25)	0.132
WHtR	0.53 (0.49, 0.57)	0.53 (0.49, 0.57)	0.53 (0.49, 0.58)	0.54 (0.49, 0.58)	<0.001
WHR	0.89 (0.85, 0.94)	0.89 (0.85, 0.94)	0.89 (0.85, 0.94)	0.9 (0.85, 0.94)	0.202
FPG, mmol/L	5.08 (4.68, 5.50)	5.11 (4.69, 5.47)	5.07 (4.68, 5.50)	5.14 (4.75, 5.53)	0.023
SBP, mmHg	121.33 (111.33, 135.00)	123.00 (111.00, 137.00)	125.00 (112.67, 139.67)	126.33 (113.67, 140.67)	<0.001
DBP, mmHg	77.33 (70.33, 85.00)	77.00 (69.67, 85.33)	76.67 (70.00, 85.00)	77.33 (70.00, 85.67)	0.370
TC, mmol/L	4.27 (3.70, 4.97)	4.28 (3.75, 4.90)	4.37 (3.80, 4.96)	4.42 (3.84, 5.08)	<0.001
TG, mmol/L	1.35 (0.95, 1.96)	1.34 (0.96, 1.96)	1.35 (0.96, 1.92)	1.39 (1.00, 2.02)	0.066
HDL-C, mmol/L	1.07 (0.92, 1.24)	1.09 (0.94, 1.26)	1.08 (0.93, 1.25)	1.08 (0.93, 1.26)	0.207
LDL-C, mmol/L	2.46 (2.00, 3.01)	2.47 (2.04, 2.99)	2.57 (2.07, 3.08)	2.57 (2.12, 3.13)	<0.001
Family history of diabetes (%)	237 (11.79)	257(12.69)	216(10.70)	186(9.30)	<0.001
Hypertension (%)	667 (33.17)	705 (34.81)	743 (36.80)	795 (39.75)	<0.001
Dyslipidemia (%)	1004 (53.52)	895 (49.86)	909 (52.21)	934 (52.80)	0.140

Abbreviations: DII, dietary inflammatory index; BMI, body mass index; WC, waist circumference; WHtR, waist-to-height ratio; WHR, waist-to-hip ratio; FPG, fasting plasma glucose; SBP, systolic blood pressure; DBP, diastolic blood pressure; TC, total cholesterol; TG, triglycerides; HDL-C, high-density lipoprotein cholesterol; LDL-C, low-density lipoprotein cholesterol. Note: Variables are presented as the median (interquartile range) or frequency (percentage).

Table S4. Baseline characteristics of the study participants by gender.

Variables	Male<br>(*n* = 3241)	Female<br>(*n* = 4814)	*P* value
Age, years	59.00 (49.00, 67.00)	54.00 (46.00, 63.00)	<0.001
High school and above (%)	495 (15.32)	306 (6.36)	<0.001
Married/cohabiting (%)	2901 (89.56)	4377 (91.00)	0.032
Mean individual monthly income ≤1000 CNY (%)	2906 (90.53)	4436 (92.98)	<0.001
Current smoking (%)	1583 (48.84)	12 (0.25)	<0.001
Alcohol drinking (%)	933 (28.79)	40 (0.83)	<0.001
Sleep duration, h/day	8.00 (7.00, 9.50)	8.00 (7.00, 9.00)	0.352
Ideal physical activity (%)	1737 (53.59)	4576 (95.06)	<0.001
DII score	2.39 (1.58, 2.84)	2.66 (1.93, 2.98)	<0.001
BMI, kg/m^2	24.08 (21.79, 26.46)	25.10 (22.70, 27.64)	<0.001
WC, cm	84.75 (77.50, 91.50)	84.25 (77.25, 91.00)	0.031
WHtR	0.51 (0.47, 0.55)	0.54 (0.50, 0.59)	<0.001
WHR	0.91 (0.86, 0.95)	0.89 (0.84, 0.93)	<0.001
FPG, mmol/L	5.12 (4.705, 5.52)	5.09 (4.70, 5.49)	0.191
SBP, mmHg	124.00 (113.33, 137.33)	123.33 (111.33, 138.67)	0.124
DBP, mmHg	77.00 (70.00, 85.33)	77.00 (70.00, 85.00)	0.985
TC, mmol/L	4.21 (3.69, 4.82)	4.43 (3.83, 5.09)	<0.001
TG, mmol/L	1.31 (0.95, 1.87)	1.39 (0.98, 2.01)	<0.001
HDL-C, mmol/L	1.03 (0.89, 1.20)	1.12 (0.96, 1.28)	<0.001
LDL-C, mmol/L	2.47 (2.02, 2.96)	2.56 (2.08, 3.10)	<0.001
Family history of diabetes (%)	333 (10.27)	563 (11.70)	0.120
Hypertension (%)	1089 (33.60)	1821 (37.83)	<0.001
Dyslipidemia (%)	1651 (58.26)	2091 (48.10)	<0.001
Obesity (%)	396 (12.22)	1040 (21.60)	<0.001
Menopause (%)	-	3038 (63.11)	-
Number of pregnancies ≥ 3 (%)	-	3090 (64.33)	-

Abbreviations: DII, dietary inflammatory index; BMI, body mass index; WC, waist circumference; WHtR, waist-to-height ratio; WHR, waist-to-hip ratio; FPG, fasting plasma glucose; SBP, systolic blood pressure; DBP, diastolic blood pressure; TC, total cholesterol; TG, triglycerides; HDL-C, high-density lipoprotein cholesterol; LDL-C, low-density lipoprotein cholesterol. Note: Variables are presented as the median (interquartile range) or frequency (percentage).

Table S5. Dietary intake of each food parameter grouped by T2DM status.

food parameters	Total<br>(*n* = 8055)	T2DM<br>(*n* = 1034)	Non-T2DM (*n* = 7021)	*P* value
Energy, kcal	1259.86 (997.76, 1526.70)	1273.20 (1018.18, 1543.89)	1257.72 (993.71, 1524.70)	0.243
Carbohydrate, g	232.97 (184.17, 286.53)	234.88 (191.21, 289.20)	232.68 (183.35, 286.00)	0.040
Protein, g	45.26 (35.43, 57.22)	44.87 (35.09, 57.62)	45.35 (35.50, 57.16)	0.986
Total fat, g	15.10 (9.39, 23.28)	14.26 (9.12, 22.49)	15.23 (9.43, 23.41)	0.058
Fiber, g,	6.86 (5.27, 8.80)	6.84 (5.18, 8.85)	6.87 (5.29, 8.79)	0.709
Cholesterol, mg	266.81 (86.47, 560.86)	240.22 (61.11, 560.33)	273.82 (87.39, 560.93)	0.056
Saturated fat, g	5.69 (3.47, 8.61)	5.48 (3.37, 8.35)	5.74 (3.49, 8.62)	0.133
MUFA, g	4.29 (2.51, 6.87)	4.00 (2.41, 6.50)	4.32 (2.54, 6.90)	0.052
PUFA, g	2.39 (1.49, 4.18)	2.25 (1.41, 3.93)	2.41 (1.50, 4.20)	0.043
Niacin, mg	6.71 (5.22, 8.41)	6.71 (5.16, 8.49)	6.71 (5.23, 8.40)	0.788
Thiamin, mg	0.72 (0.57, 0.88)	0.73 (0.57, 0.89)	0.72 (0.57, 0.87)	0.398
Riboflavin, mg	0.45 (0.34, 0.58)	0.45 (0.34, 0.59)	0.45 (0.34, 0.58)	0.706

Vitamin A, μg	231.74 (148.51, 347.49)	226.76 (141.81, 346.44)	232.58 (149.77, 347.98)	0.174
Vitamin C, mg	51.54 (32.95, 71.48)	50.80 (32.35, 69.92)	51.62 (33.07, 71.66)	0.371
Vitamin E, mg	6.19 (4.51, 8.73)	6.08 (4.40, 8.38)	6.21 (4.53, 8.77)	0.086
Fe, mg	14.86 (11.79, 18.10)	14.95 (11.86, 18.30)	14.85 (11.78, 18.06)	0.366
Mg, mg	215.58 (168.37, 265.78)	216.46 (167.02, 267.76)	215.41 (168.6, 265.54)	0.819
Zn, mg	5.68 (4.49, 7.07)	5.65 (4.49, 7.14)	5.69 (4.49, 7.06)	0.777
Se, μg	34.28 (26.67, 42.71)	34.25 (26.58, 43.3)	34.28 (26.69, 42.60)	0.981
Folic acid, μg	121.97 (80.67, 172.44)	120.00 (76.35, 170.68)	122.18 (81.28, 172.56)	0.098
β-Carotene, μg	1792.75 (1127.31, 2168.52)	1792.75 (1127.31, 2168.52)	1792.75 (1127.31, 2168.52)	0.980
Isoflavones, mg	4.28 (1.52, 9.93)	4.27 (1.10, 8.54)	4.29 (1.53, 9.94)	0.015
Anthocyanidins, mg	3.08 (1.41, 8.64)	3.03 (1.21, 8.52)	3.11 (1.41, 8.71)	0.033
Alcohol, g	0.00 (0.00, 0.00)	0.00 (0.00, 0.00)	0.00 (0.00, 0.00)	0.810

Abbreviations: T2DM, type 2 diabetes mellitus; MUFA, monounsaturated fatty acids; PUFA, polyunsaturated fatty acids. Note: Variables are presented as the median (interquartile range).

Table S6. Food parameter-specific DII scores grouped by overall DII quartiles among participants.

**food parameters**	**Q1 (*n* = 2011)**	**Q2 (*n* = 2025)**	**Q3 (*n* = 2019)**	**Q4 (*n* = 2000)**	***P* value**
Energy	−0.14 (−0.17, −0.06)	−0.17 (−0.18, −0.16)	−0.18 (−0.18, −0.18)	−0.18 (−0.18, −0.18)	<0.001
Carbohydrate	0.03 (−0.05, 0.08)	−0.05 (−0.09, 0.03)	−0.09 (−0.1, −0.06)	−0.09 (−0.1, −0.03)	<0.001
Protein	−0.01 (−0.02, 0.00)	−0.02 (−0.02, −0.02)	−0.02 (−0.02, −0.02)	−0.02 (−0.02, −0.02)	<0.001
Total fat	−0.29 (−0.3, −0.27)	−0.3 (−0.3, −0.29)	−0.30 (−0.30, −0.30)	−0.30 (−0.30, −0.30)	<0.001
Fiber	0.61 (0.55, 0.63)	0.65 (0.64, 0.65)	0.66 (0.66, 0.66)	0.66 (0.65, 0.66)	<0.001
Cholesterol	0.06 (−0.11, 0.11)	−0.01 (−0.11, 0.11)	−0.11 (−0.11, 0.05)	−0.11 (−0.11, 0.11)	<0.001
Saturated fat	−0.37 (−0.37, −0.35)	−0.37 (−0.37, −0.37)	−0.37 (−0.37, −0.37)	−0.37 (−0.37, -0.37)	<0.001
MUFA	0.01 (0.01, 0.01)	0.01 (0.01, 0.01)	0.01 (0.01, 0.01)	0.01 (0.01, 0.01)	<0.001
PUFA	0.33 (0.27, 0.34)	0.34 (0.33, 0.34)	0.34 (0.34, 0.34)	0.34 (0.34, 0.34)	<0.001
Niacin	0.21 (0.19, 0.21)	0.22 (0.21, 0.22)	0.23 (0.22, 0.23)	0.23 (0.22, 0.23)	<0.001
Thiamin	0.07 (0.06, 0.08)	0.08 (0.08, 0.09)	0.09 (0.09, 0.09)	0.09 (0.08, 0.09)	<0.001
Riboflavin	0.06 (0.05, 0.06)	0.06 (0.06, 0.06)	0.06 (0.06, 0.06)	0.06 (0.06, 0.06)	<0.001
Vitamin A	0.31 (0.27, 0.33)	0.34 (0.31, 0.35)	0.36 (0.34, 0.36)	0.35 (0.33, 0.36)	<0.001
Vitamin C	0.24 (0.09, 0.36)	0.35 (0.3, 0.38)	0.4 (0.38, 0.41)	0.39 (0.37, 0.41)	<0.001
Vitamin E	−0.42 (−0.42, −0.18)	0.29 (0.11, 0.37)	0.41 (0.39, 0.42)	0.42 (0.42, 0.42)	<0.001
Fe	0.03 (0.02, 0.03)	0.01 (0.00, 0.02)	−0.01 (−0.02, 0.01)	−0.01 (−0.02, 0.01)	<0.001
Mg	0.01 (−0.13, 0.12)	0.21 (0.15, 0.27)	0.33 (0.27, 0.36)	0.33 (0.25, 0.38)	<0.001
Zn	0.20 (0.06, 0.26)	0.29 (0.26, 0.30)	0.31 (0.30, 0.31)	0.31 (0.30, 0.31)	<0.001
Se	0.12 (0.07, 0.14)	0.15 (0.13, 0.16)	0.17 (0.15, 0.18)	0.17 (0.15, 0.18)	<0.001
Folic acid	0.12 (0.03, 0.17)	0.18 (0.16, 0.19)	0.19 (0.18, 0.19)	0.19 (0.18, 0.19)	<0.001
β-Carotene	0.31 (0.04, 0.43)	0.42 (0.36, 0.47)	0.50 (0.46, 0.53)	0.50 (0.42, 0.53)	<0.001
Isoflavones	−0.59 (−0.59, −0.59)	−0.59 (−0.59, −0.59)	−0.59 (−0.59, −0.59)	0.59 (0.36, 0.59)	<0.001
Anthocyanidins	0.04 (0.01, 0.07)	0.06 (0.04, 0.07)	0.07 (0.06, 0.08)	0.07 (0.06, 0.08)	<0.001
Alcohol	0.28 (0.28, 0.28)	0.28 (0.28, 0.28)	0.28 (0.28, 0.28)	0.28 (0.28, 0.28)	<0.001

Abbreviations: T2DM, type 2 diabetes mellitus; MUFA, monounsaturated fatty acids; PUFA, polyunsaturated fatty acids. Note: Variables are presented as the median (interquartile range).

Table S7. Interactions and joint associations of obesity metrics and binary DII groups on the risk of T2DM among males.

Obesity groups	DII groups	Joint association		Multiplicative interaction		Additive interaction	
		HR (95% CI)	*P* value	HR (95% CI)	*P* value	RERI (95%CI)	AP (95%CI)
BMI							
Normal	Low	1					
Normal	High	0.67 (0.47, 0.95)	0.024				
Obesity	Low	0.82 (0.54, 1.26)	0.365				
Obesity	High	1.16 (0.64, 2.11)	0.627	2.12 (1.13, 3.98)	0.019	0.68 (0.12, 1.53)	0.58 (−0.05, 0.93)
WHtR							
Normal	Low	1					
Normal	High	0.57 (0.32, 1.04)	0.069				
Obesity	Low	1.49 (1.07, 2.08)	0.018				
Obesity	High	1.35 (0.88, 2.06)	0.168	1.58 (0.80, 3.11)	0.186	0.29 (−0.41, 0.88)	0.21 (−0.35, 0.55)
WHR							
Normal	Low	1					
Normal	High	0.47 (0.26, 0.86)	0.013				
Obesity	Low	1.29 (0.96, 1.74)	0.092				
Obesity	High	1.31 (0.88, 1.95)	0.190	2.14 (1.09, 4.18)	0.027	0.54 (−0.03, 1.09)	0.41 (−0.06, 0.71)
WC							
Normal	Low	1					
Normal	High	0.49 (0.31, 0.79)	0.029				
Obesity	Low	1.71 (1.26, 2.32)	0.001				
Obesity	High	2.03 (1.33, 3.09)	0.001	2.41 (1.34, 4.34)	0.003	0.83 (0.11, 1.76)	0.41 (0.02, 0.62)

Abbreviations: T2DM, type 2 diabetes mellitus; DII, dietary inflammatory index; HR, hazard ratio; CI, confidence interval; BMI, body mass index; WC, waist circumference; WHtR, waist-to-height ratio; WHR, waist-to-hip ratio; RERI, relative excess risk due to interaction; AP, attributable proportion due to interaction. Adjusted for: age, gender, educational level, marital status, average monthly income, smoking status, drinking status, physical activity, sleep duration, BMI status, family history of diabetes, energy intake, hypertension, dyslipidemia, and fasting plasma glucose. Note: The reference category for joint association analysis is participants with normal BMI/WHR/WC/WHtR and a low DII score. Interpretation of additive interaction indices: RERI quantifies the excess disease risk attributable specifically to the interaction between two exposures (such as high DII and obesity), beyond the sum of their individual effects. AP estimates the proportion of disease risk in the doubly exposed group that is due to this interaction. An additive interaction is suggested when the 95% confidence interval for both RERI and AP does not include 0.

Table S8. Interactions and joint associations of dyslipidemia types and binary DII groups on the risk of T2DM among males.

Dyslipidemia groups	DII groups	Joint association		Multiplicative interaction		Additive interaction	
		HR (95% CI)	*P* value	HR (95% CI)	*P* value	RERI (95%CI)	AP (95%CI)
High TC							
No	Low	1					
No	High	0.78 (0.57, 1.05)	0.100				
Yes	Low	0.63 (0.31, 1.26)	0.189				
Yes	High	1.57 (0.38, 6.44)	0.540	3.23 (0.66, 15.7)	0.146	1.17 (−0.19, 6.03)	0.74 (−1.76, 2.07)
High TG							
No	Low	1					
No	High	0.72 (0.52, 1.02)	0.061				
Yes	Low	1.19 (0.88, 1.60)	0.258				
Yes	High	1.39 (0.80, 2.44)	0.248	1.62 (0.83, 3.16)	0.156	0.48 (−0.24, 1.53)	0.35 (−0.45, 0.59)
Low HDL-C							
No	Low	1					
No	High	0.81 (0.53, 1.26)	0.359				
Yes	Low	1.01 (0.68, 1.49)	0.969				
Yes	High	0.80 (0.48, 1.34)	0.412	0.98 (0.55, 1.74)	0.942	−0.02 (−0.61, 0.46)	−0.02 (−0.97, 0.49)
High LDL-C							
No	Low	1					
No	High	0.80 (0.58, 1.09)	0.153				
Yes	Low	0.66 (0.27, 1.62)	0.363				
Yes	High	0.40 (0.06, 2.88)	0.368	0.76 (0.09, 6.78)	0.806	−0.06 (−1.13, 2.46)	−0.14 (−22.85, 12.51)

Abbreviations: T2DM, type 2 diabetes mellitus; DII, dietary inflammatory index; HR, hazard ratio; CI, confidence interval; High TC, hypercholesterolemia; High TG, hypertriglyceridemia; Low HDL-C, low high-density lipoprotein cholesterol; High LDL-C, high low-density lipoprotein cholesterol; RERI, relative excess risk due to interaction; AP, attributable proportion due to interaction. Adjusted for: age, gender, educational level, marital status, average monthly income, smoking status, drinking status, physical activity, sleep duration, BMI status, family history of diabetes, energy intake, hypertension, dyslipidemia, and fasting plasma glucose. Note: The reference category for joint

association analysis is participants without the specific dyslipidemia and a low DII score. Interpretation of additive interaction indices: RERI quantifies the excess disease risk attributable specifically to the interaction between two exposures (such as high DII and high TC), beyond the sum of their individual effects. AP estimates the proportion of disease risk in the doubly exposed group that is due to this interaction. An additive interaction is suggested when the 95% confidence interval for both RERI and AP does not include 0.

Table S9. Interactions and joint associations of obesity metrics and binary DII groups on the risk of T2DM among females.

Obesity groups	DII groups	Joint association		Multiplicative interaction		Additive interaction	
		**HR (95% CI)**	***P* value**	**HR (95% CI)**	***P* value**	**RERI (95%CI)**	**AP (95%CI)**
BMI							
Normal	Low	1					
Normal	High	1.14 (0.91, 1.43)	0.259				
Obesity	Low	0.66 (0.46, 0.93)	0.019				
Obesity	High	0.73 (0.49, 1.09)	0.120	0.98 (0.67, 1.41)	0.893	−0.07 (−0.43, 0.26)	−0.09 (−0.75, 0.28)
WHtR							
Normal	Low	1					
Normal	High	1.12 (0.67, 1.87)	0.669				
Obesity	Low	1.29 (0.92, 1.82)	0.141				
Obesity	High	1.36 (0.94, 1.96)	0.104	0.94 (0.54, 1.61)	0.816	−0.06 (−0.91, 0.43)	−0.04 (−0.59, 0.36)
WHR							
Normal	Low	1					
Normal	High	0.86 (0.52, 1.44)	0.577				
Obesity	Low	1.31 (0.97, 1.77)	0.080				
Obesity	High	1.43 (1.03, 1.98)	0.033	1.26 (0.73, 2.17)	0.400	0.26 (−0.41, 0.69)	0.18 (−0.26, 0.50)
WC							
Normal	Low	1					
Normal	High	1.09 (0.79, 1.50)	0.600				
Obesity	Low	1.56 (1.20, 2.04)	0.001				
Obesity	High	1.80 (1.33, 2.43)	<0.001	1.06 (0.72, 1.55)	0.769	0.15 (−0.40, 0.64)	0.08 (−0.24, 0.31)

Abbreviations: T2DM, type 2 diabetes mellitus; DII, dietary inflammatory index; HR, hazard ratio; CI, confidence interval; BMI, body mass index; WC, waist circumference; WHtR, waist-to-height ratio; WHR, waist-to-hip ratio; RERI, relative excess risk due to interaction; AP, attributable proportion due to interaction. Adjusted for: age, gender, educational level, marital status, average monthly income, smoking status, drinking status, physical activity, sleep duration, BMI status, family history of diabetes, energy intake, hypertension, dyslipidemia, and fasting plasma glucose. Note: The reference category for joint association analysis is participants with normal BMI/WHR/WC/WHtR and a low DII score. Interpretation of additive interaction indices: RERI quantifies the excess disease risk attributable specifically to the interaction between two exposures (such as high DII and obesity), beyond the sum of their individual effects. AP estimates the proportion of disease risk in the doubly exposed group that is due to this interaction. An additive interaction is suggested when the 95% confidence interval for both RERI and AP does not include 0.

Table S10. Interactions and joint associations of dyslipidemia types and binary DII groups on the risk of T2DM among females.

Dyslipidemia groups	DII groups	Joint association		Multiplicative interaction		Additive interaction	
		HR (95% CI)	*P* value	HR (95% CI)	*P* value	RERI (95%CI)	AP (95%CI)
High TC							
No	Low	1					
No	High	1.13 (0.93, 1.37)	0.224				
Yes	Low	1.02 (0.68, 1.54)	0.923				
Yes	High	1.20 (0.68, 2.11)	0.547	1.04 (0.52, 2.09)	0.913	0.05 (−0.70, 1.00)	0.04 (−1.10, 0.36)
High TG							
No	Low	1					
No	High	1.21 (0.97, 1.50)	0.090				
Yes	Low	1.26 (0.97, 1.62)	0.081				
Yes	High	1.22 (0.88, 1.70)	0.228	0.81 (0.55, 1.19)	0.280	−0.24 (−0.74, 0.25)	−0.20 (−0.75, 0.12)
Low HDL-C							
No	Low	1					
No	High	1.26 (0.99, 1.59)	0.059				
Yes	Low	1.05 (0.80, 1.38)	0.733				
Yes	High	1.03 (0.74, 1.42)	0.883	0.78 (0.54, 1.12)	0.175	−0.28 (−0.73, 0.12)	−0.27 (−0.82, 0.06)
High LDL-C							
No	Low	1					
No	High	1.12 (0.92, 1.36)	0.274				
Yes	Low	0.59 (0.32, 1.09)	0.094				
Yes	High	0.95 (0.50, 1.81)	0.890	1.44 (0.59, 3.48)	0.422	0.24 (−0.46, 1.13)	0.25 (−1.08, 0.61)

Abbreviations: T2DM, type 2 diabetes mellitus; DII, dietary inflammatory index; HR, hazard ratio; CI, confidence interval; High TC, hypercholesterolemia; High TG, hypertriglyceridemia; Low HDL-C, low high-density lipoprotein cholesterol; High LDL-C, high low-density lipoprotein cholesterol; RERI, relative excess risk due to interaction; AP, attributable proportion due to interaction. Adjusted for: age, gender, educational level, marital status, average monthly income, smoking status, drinking status, physical activity, sleep duration, BMI status, family history of diabetes, energy intake, hypertension, dyslipidemia, and fasting plasma glucose. Note: The reference category for joint

association analysis is participants without the specific dyslipidemia and a low DII score. Interpretation of additive interaction indices: RERI quantifies the excess disease risk attributable specifically to the interaction between two exposures (such as high DII and high TC), beyond the sum of their individual effects. AP estimates the proportion of disease risk in the doubly exposed group that is due to this interaction. An additive interaction is suggested when the 95% confidence interval for both RERI and AP does not include 0.

Table S11. Subgroup analysis of the association between DII level (category and per 1-SD) and risk of T2DM.

**Variable**		**DII level**	**HR (95%CI)**	***P* value**	***P* for interaction**
Age, years					0.662
	<60	Q1	1		
		Q2	1.24 (0.96, 1.61)	0.102	
		Q3	1.30 (0.96, 1.75)	0.089	
		Q4	1.24 (0.92, 1.67)	0.155	
		Per 1-SD	1.10 (0.99, 1.23)	0.088	
	≥60	Q1	1		
		Q2	0.93 (0.67, 1.28)	0.661	
		Q3	1.03 (0.74, 1.43)	0.858	
		Q4	1.09 (0.79, 1.50)	0.608	
		Per 1-SD	1.03 (0.91, 1.17)	0.610	
Alcohol drinking					0.255
	No	Q1	1		
		Q2	1.12 (0.89, 1.39)	0.332	
		Q3	1.20 (0.95, 1.52)	0.122	
		Q4	1.21 (0.96, 1.53)	0.098	
		Per 1-SD	1.10 (1.01, 1.20)	0.034	
	Yes	Q1	1		
		Q2	0.91 (0.54, 1.53)	0.723	
		Q3	0.61 (0.29, 1.26)	0.183	
		Q4	0.73 (0.34, 1.57)	0.422	
		Per 1-SD	0.82 (0.65, 1.03)	0.089	
Smoking status					0.342
	Never smoked	Q1	1		
		Q2	1.17 (0.91, 1.51)	0.210	
		Q3	1.23 (0.94, 1.60)	0.133	
		Q4	1.27 (0.98, 1.65)	0.071	
		Per 1-SD	1.11 (1.00, 1.23)	0.045	
	Current smoking	Q1	1		
		Q2	0.85 (0.57, 1.27)	0.427	
		Q3	0.82 (0.50, 1.34)	0.432	
		Q4	0.68 (0.40, 1.16)	0.156	
		Per 1-SD	0.90 (0.76, 1.08)	0.258	
	Quit smoking	Q1	1		
		Q2	0.93 (0.48, 1.83)	0.842	

	Q3	1.09 (0.52, 2.30)	0.823	
	Q4	1.12 (0.55, 2.28)	0.746	
	Per 1-SD	1.04 (0.80, 1.36)	0.768	
Ideal physical activity				0.522
No	Q1	1		
	Q2	1.01 (0.66, 1.55)	0.956	
	Q3	0.82 (0.48, 1.39)	0.464	
	Q4	0.94 (0.57, 1.55)	0.806	
	Per 1-SD	0.96 (0.79, 1.16)	0.645	
Yes	Q1	1		
	Q2	1.10 (0.87, 1.38)	0.436	
	Q3	1.21 (0.95, 1.54)	0.129	
	Q4	1.22 (0.96, 1.54)	0.108	
	Per 1-SD	1.09 (1.00, 1.20)	0.063	
Hypertension				0.964
No	Q1	1		
	Q2	1.17 (0.89, 1.54)	0.261	
	Q3	1.17 (0.86, 1.58)	0.325	
	Q4	1.14 (0.84, 1.55)	0.397	
	Per 1-SD	1.05 (0.93, 1.18)	0.431	
Yes	Q1	1		
	Q2	0.98 (0.73, 1.32)	0.914	
	Q3	1.09 (0.79, 1.50)	0.596	
	Q4	1.15 (0.85, 1.56)	0.365	
	Per 1-SD	1.08 (0.96, 1.21)	0.225	
Family history of diabetes				0.746
No	Q1	1		
	Q2	1.00 (0.73, 1.36)	0.995	
	Q3	1.07 (0.76, 1.50)	0.698	
	Q4	0.96 (0.70, 1.33)	0.820	
	Per 1-SD	1.01 (0.90, 1.15)	0.818	
Yes	Q1	1		
	Q2	1.22 (0.76, 1.96)	0.404	
	Q3	1.00 (0.58, 1.72)	0.993	
	Q4	0.96 (0.56, 1.63)	0.870	
	Per 1-SD	0.94 (0.77, 1.15)	0.535	

Abbreviations: T2DM, type 2 diabetes mellitus; DII, dietary inflammatory index; HR, hazard ratio; CI, confidence interval; SD, standard deviation. Adjusted for: age, gender, educational level, marital status, average monthly income, smoking status, drinking status, physical activity, sleep duration, BMI status, family history of diabetes, energy intake, hypertension, dyslipidemia, and fasting plasma glucose.

Table S12. Sensitivity analysis of the association between DII levels and T2DM: Exclusion of individuals who developed T2DM within the first two years of follow-up.

Quartile group of DII		Model 1		Model 2		Model 3	
		**HR (95% CI)**	***P* value**	**HR (95% CI)**	***P* value**	**HR (95% CI)**	***P* value**
Total							
	Q1	1		1		1	
	Q2	1.08 (0.90, 1.30)	0.413	1.05 (0.88, 1.26)	0.592	1.08 (0.89, 1.32)	0.440
	Q3	1.18 (0.99, 1.42)	0.066	1.09 (0.91, 1.31)	0.352	1.13 (0.91, 1.41)	0.277
	Q4	1.19 (1.00, 1.42)	0.056	1.05 (0.88, 1.26)	0.589	1.15 (0.93, 1.43)	0.198
	P trend	0.042		0.425		0.193	
	Per 1-SD increase	1.07 (1.00, 1.13)	0.039	1.02 (0.96, 1.09)	0.480	1.08 (0.99, 1.18)	0.073
Male							
	Q1	1		1		1	
	Q2	1.02 (0.79, 1.33)	0.877	0.98 (0.76, 1.28)	0.903	0.96 (0.71, 1.29)	0.779
	Q3	1.05 (0.79, 1.39)	0.738	0.98 (0.74, 1.31)	0.897	1.02 (0.72, 1.43)	0.934
	Q4	0.87 (0.65, 1.17)	0.358	0.79 (0.58, 1.07)	0.123	0.82 (0.57, 1.18)	0.285
	P trend	0.662		0.275		0.494	
	Per 1-SD increase	0.97 (0.89, 1.06)	0.499	0.94 (0.86, 1.03)	0.161	0.94 (0.82, 1.07)	0.344
Female							
	Q1	1		1		1	
	Q2	1.18 (0.91, 1.53)	0.206	1.15 (0.89, 1.49)	0.298	1.19 (0.90, 1.57)	0.227
	Q3	1.38 (1.08, 1.76)	0.010	1.23 (0.96, 1.58)	0.096	1.22 (0.91, 1.63)	0.191
	Q4	1.50 (1.18, 1.90)	0.001	1.26 (0.99, 1.62)	0.061	1.36 (1.03, 1.81)	0.031
	P trend	0.002		0.057		0.056	
	Per 1-SD increase	1.19 (1.09, 1.29)	<0.001	1.11 (1.02, 1.22)	0.019	1.20 (1.07, 1.34)	0.002

Abbreviations: T2DM, type 2 diabetes mellitus; DII, dietary inflammatory index; HR, hazard ratio; CI, confidence interval; SD, standard deviation. Model 1: Unadjusted. Model 2: Adjusted for age, gender, educational level, marital status, and average monthly income. Model 3: Adjusted for model 2 variables and smoking status, drinking status, physical activity, sleep duration, BMI status, family history of diabetes, energy intake, hypertension, dyslipidemia, and fasting plasma glucose. Note: For single-gender dataset analyses, gender was not included as a covariate.

Table S13. Sensitivity analysis of the association between DII levels and T2DM: Imputing missing values in variables using multiple imputation.

Quartile group of DII		Model 1		Model 2		Model 3	
		HR (95% CI)	*P* value	HR (95% CI)	*P* value	HR (95% CI)	*P* value
Total							
	Q1	1		1		1	
	Q2	1.03 (0.86, 1.23)	0.751	1.01 (0.84, 1.20)	0.938	1.08 (0.89, 1.31)	0.420
	Q3	1.19 (1.00, 1.42)	0.045	1.10 (0.93, 1.31)	0.277	1.20 (0.98, 1.48)	0.083
	Q4	1.20 (1.01, 1.42)	0.038	1.07 (0.89, 1.27)	0.478	1.19 (0.97, 1.46)	0.093
	P trend	0.036		0.386		0.076	
	Per 1-SD increase	1.07 (1.01, 1.13)	0.030	1.02 (0.96, 1.09)	0.439	1.07 (0.99, 1.16)	0.095
Male							
	Q1	1		1		1	
	Q2	1.00 (0.77, 1.29)	0.980	0.96 (0.75, 1.24)	0.771	0.99 (0.75, 1.32)	0.956
	Q3	1.11 (0.85, 1.45)	0.450	1.04 (0.79, 1.36)	0.800	1.09 (0.79, 1.50)	0.620
	Q4	0.88 (0.66, 1.18)	0.393	0.80 (0.60, 1.08)	0.142	0.91 (0.65, 1.28)	0.600
	P trend	0.796		0.358		0.892	
	Per 1-SD increase	1.00 (0.90, 1.10)	0.645	0.95 (0.87, 1.03)	0.223	0.99 (0.87, 1.11)	0.802
Female							
	Q1	1		1		1	
	Q2	1.10 (0.85, 1.41)	0.476	1.07 (0.83, 1.38)	0.593	1.15 (0.88, 1.50)	0.303
	Q3	1.33 (1.06, 1.69)	0.016	1.21 (0.95, 1.53)	0.122	1.28 (0.97, 1.69)	0.076
	Q4	1.48 (1.17, 1.85)	0.001	1.26 (1.00, 1.59)	0.053	1.37 (1.05, 1.79)	0.022
	P trend	0.003		0.073		0.034	
	Per 1-SD increase	1.17 (1.08, 1.27)	<0.001	1.10 (1.01, 1.20)	0.025	1.14 (1.03, 1.27)	0.013

Abbreviations: T2DM, type 2 diabetes mellitus; DII, dietary inflammatory index; HR, hazard ratio; CI, confidence interval; SD, standard deviation. Model 1: Unadjusted. Model 2: Adjusted for age, gender, educational level, marital status, and average monthly income. Model 3: Adjusted for model 2 variables and smoking status, drinking status, physical activity, sleep duration, BMI status, family history of diabetes, energy intake, hypertension, dyslipidemia, and fasting plasma glucose. Note: For single-gender dataset analyses, gender was not included as a covariate.

Table S14. Sensitivity analysis of the association between DII levels and T2DM: Substituting BMI with alternative adiposity metrics (WHtR, WHR, or WC) in Model 3.

Quartile group of DII	Adjust for WHtR		Adjust for WHR		Adjust for WC	
	**HR (95% CI)[a]**	***P* value**	**HR (95% CI)[b]**	***P* value**	**HR (95% CI)[c]**	***P* value**
Total						
Q1	1		1		1	
Q2	1.06 (0.87, 1.29)	0.583	1.04 (0.85, 1.27)	0.733	1.06 (0.87, 1.30)	0.552
Q3	1.12 (0.90, 1.39)	0.315	1.09 (0.87, 1.35)	0.461	1.13 (0.91, 1.41)	0.269
Q4	1.13 (0.91, 1.41)	0.256	1.12 (0.90, 1.38)	0.320	1.13 (0.91, 1.41)	0.258
P trend	0.253		0.358		0.235	
Per 1-SD increase	1.05 (0.97, 1.15)	0.222	1.05 (0.97, 1.14)	0.263	1.06 (0.97, 1.15)	0.193
Male						
Q1	1		1		1	
Q2	0.93 (0.69, 1.26)	0.640	0.94 (0.69, 1.26)	0.662	0.97 (0.72, 1.31)	0.860
Q3	1.01 (0.72, 1.43)	0.936	1.00 (0.71, 1.42)	0.987	1.07 (0.76, 1.51)	0.702
Q4	0.80 (0.55, 1.15)	0.219	0.81 (0.56, 1.17)	0.259	0.81 (0.56, 1.17)	0.256
P trend	0.416		0.440		0.555	
Per 1-SD increase	0.93 (0.82, 1.06)	0.273	0.94 (0.82, 1.06)	0.314	0.94 (0.83, 1.07)	0.373
Female						
Q1	1		1		1	
Q2	1.17 (0.89, 1.55)	0.257	1.14 (0.86, 1.50)	0.371	1.15 (0.87, 1.51)	0.337
Q3	1.20 (0.90, 1.61)	0.221	1.16 (0.87, 1.56)	0.313	1.20 (0.90, 1.61)	0.215
Q4	1.36 (1.02, 1.80)	0.034	1.32 (0.99, 1.75)	0.055	1.35 (1.02, 1.79)	0.038
P trend	0.065		0.107		0.072	
Per 1-SD increase	1.15 (1.03, 1.29)	0.011	1.14 (1.02, 1.28)	0.017	1.16 (1.04, 1.29)	0.010

Abbreviations: T2DM, type 2 diabetes mellitus; DII, dietary inflammatory index; HR, hazard ratio; CI, confidence interval; SD, standard deviation; WC, waist circumference; WHR, waist-to-hip ratio; WHtR, waist-to-height ratio. [a]: Adjusted for age, gender, educational level, marital status, average monthly income, smoking status, drinking status, physical activity, sleep duration, WHtR status, family history of diabetes, energy intake, hypertension, dyslipidemia, and fasting plasma glucose. [b]: Adjusted for the same covariates as model [a], but with WHR status replacing WHtR status. [c]: Adjusted for the same covariates as model [a], but with WC status replacing WHtR status. Note: For single-gender dataset analyses, gender was not included as a covariate.

Table S15. Sensitivity analysis of the association between DII levels and T2DM in female participants: Further adjusted for covariates of menopausal status and pregnancies.

**Quartile group of DII**	**HR (95% CI)**	***P* value**
Q1	1	
Q2	1.19 (0.90, 1.57)	0.222
Q3	1.21 (0.90, 1.62)	0.209
Q4	1.36 (1.02, 1.80)	0.035
P trend	0.062	
Per 1-SD increase	1.16 (1.04, 1.29)	0.009

Abbreviations: T2DM, type 2 diabetes mellitus; DII, dietary inflammatory index; HR, hazard ratio; CI, confidence interval; SD, standard deviation. Adjusted for: age, gender, educational level, marital status, average monthly income, smoking status, drinking status, physical activity, sleep duration, BMI status, family history of diabetes, energy intake, hypertension, dyslipidemia, fasting plasma glucose, menopausal status, and pregnancies.
